# Supplementary material for: Genome-wide association study of lung adenocarcinoma in East Asia and comparison with a European population
Source: Nat Commun. 2023 May 26;14:3043. doi: 10.1038/s41467-023-38196-z (PMC10220065; doi:10.1038/s41467-023-38196-z)
Supplement: Supplementary file 1 — Supplementary Information [file 41467_2023_38196_MOESM1_ESM.zip › 10.1038-s41467-023-38196-z/Supplementary Information.pdf]

Supplementary Figures and Tables for "Genome-wide association study of lung adenocarcinoma in East Asia and comparison with a European population".

Supplementary Figures

1	Regional association plots for loci identified in East Asians (EA)
2	Regional association plots for the *TERT-CLPTM1L* locus in EA
3	Regional association plots for the *FOXP4* locus in EA
4	Stratified LD score regressions investigating functional enrichment and relevant tissues for LUAD heritability
5	Conditional analyses of significant TWAS loci using LCTCNS
6	Regional association plots for variants identified based on multi-ancestry meta-analysis of EA and European (EUR) samples
7	Forest plots for lung adenocarcinoma susceptibility variants identified in EA, EUR, or multi-ancestry analysis
8	Regional association plots for variants that are previously reported for EA and show no or weak association in EUR
9	Regional association plots for variants that are previously reported for EUR and show association in EA
10	Estimated heritability of lung adenocarcinoma and the genetic correlation coefficient between smokers and never-smokers, between EA and EUR
11	Testing interaction between smoking status and polygenic risk scores

Supplementary Tables:

1	Number of subjects stratified by smoking status and sex in EA
2	Meta-analysis in EA population identified 14 SNPs with genome-wide significance
3	Meta-analysis of three variants for LUAD combining the Chinese samples in Dai *et al.* and our non-overlapping EA samples
4	28 susceptibility variants identified for LUAD in EA with genome-wide significance
5	Comparing odds ratios of 28 risk variants of lung adenocarcinoma between Han Chinese and Japanese, between China mainland and others.
6	Association between new SNPs and smoking behaviors in BioBank Japan
7	Multi-ancestry meta-analysis of EA and EUR studies
8	Sample sizes of GWAS in EA and EUR studies
9	Mendelian randomization analysis

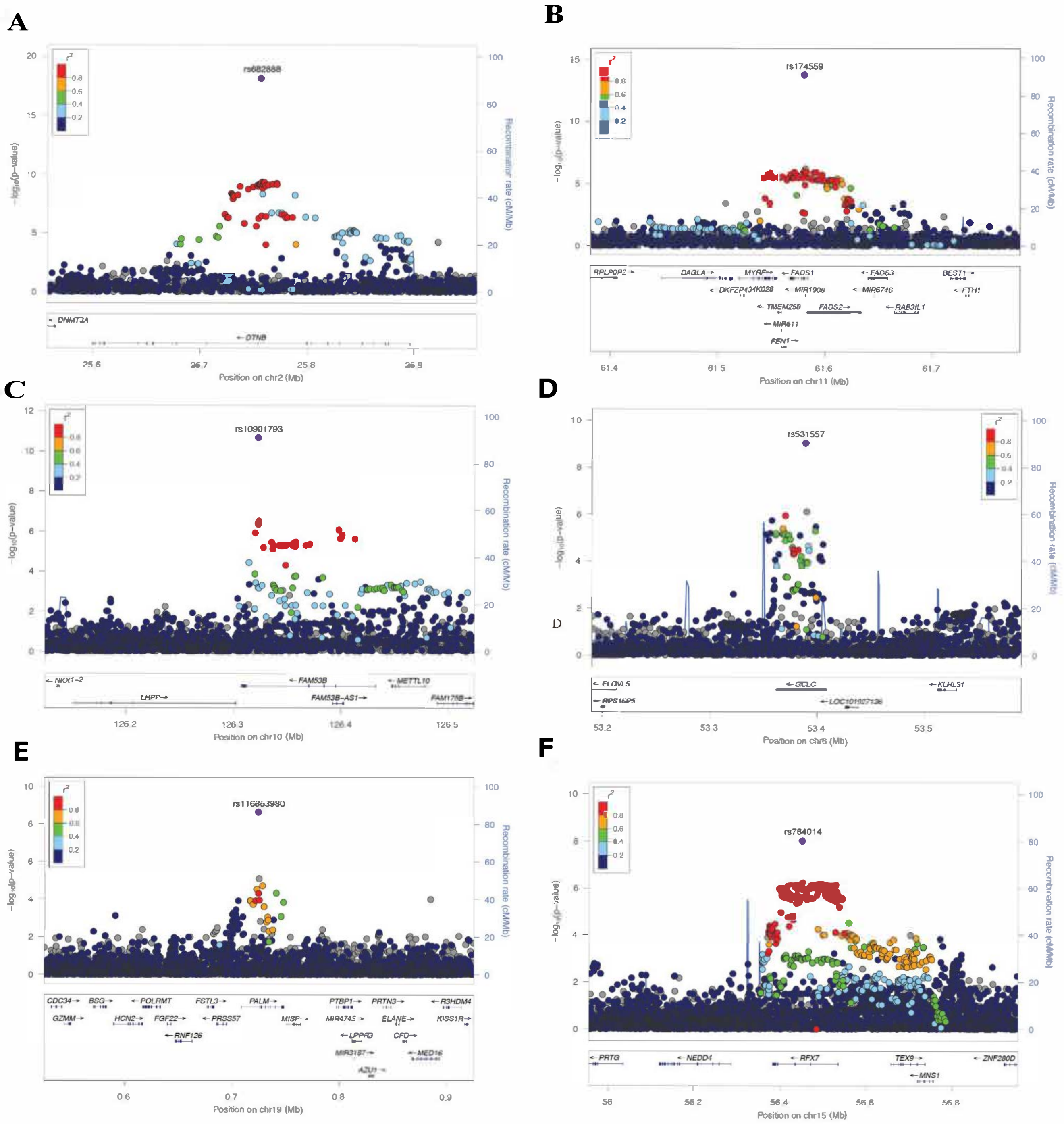
A
rs682888
−log10(p-value)
Recombination rate (cM/Mb)
DNMT3A
DTNB
Position on chr2 (Mb)
B
rs174559
RPLP0P2
DAGLA
MYRF
FADS1
FADS3
BEST1
DKFZP434K028
MIR1908
MIR6746
FTH1
TMEM258
FADS2
RAB3IL1
MIR611
FEN1
Position on chr11 (Mb)
C
rs10901793
NKX1−2
FAM53B
METTL10
LHPP
FAM53B−AS1
FAM175B
Position on chr10 (Mb)
D
rs531557
ELOVL5
GCLC
KLHL31
RPS16P5
LOC101927136
Position on chr6 (Mb)
E
rs116863980
CDC34
BSG
POLRMT
FSTL3
PALM
PTBP1
PRTN3
R3HDM4
GZMM
HCN2
FGF22
PRSS57
MISP
MIR4745
ELANE
KISS1R
RNF126
LPPR3
CFD
MIR3187
MED16
AZU1
Position on chr19 (Mb)
F
rs784014
PRTG
NEDD4
RFX7
TEX9
ZNF280D
MNS1
Position on chr15 (Mb)

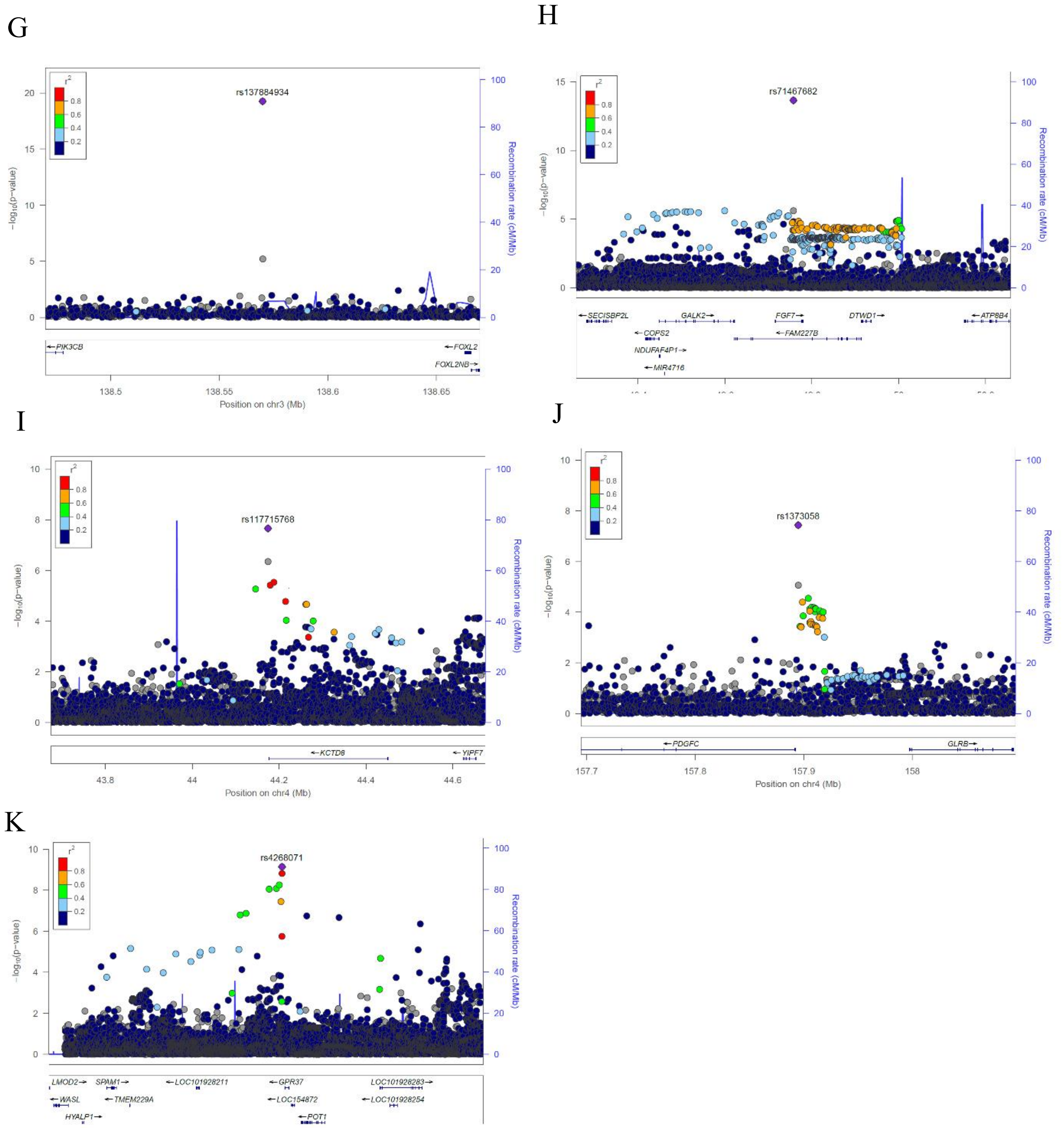

Supplementary Figure 1. Regional association plots for novel loci identified in a GWAS of lung adenocarcinoma in East Asian populations. For each panel, the p-value for the peak variant labeled in purple was from the meta-analysis of the discovery and the replication samples; other variants were based on the discovery samples. All p-values were two-sided and not adjusted for multiple testing. A: Regional association plot for rs682888 (OR=0.90, 95% CI = (0.88,0.92), P=5.96×10^{-19}). B: Regional association plot for rs174559 (OR= 0.91, 95% CI = (0.89,0.93), P=1.93×10^{-14}). C: Regional association plot for rs10901793 (OR= 1.08, 95% CI = (1.06,1.11), P=3.04×10^{-11}). D: Regional association plot for rs531557. E: Regional association plot for rs116863980 (OR= 1.21, 95% CI = (1.14,1.29), P=2.63×10^{-9}). F: Regional association plot for rs764014 (OR= 0.94, 95% CI = (0.91,0.96), P=7.73×10^{-9}). G: Regional association plot for rs137884934 (OR= 0.80, 95% CI = (0.77,0.84), P=6.21×10^{-20}). H: Regional association plot for rs71467682 (OR=0.91, 95% CI= (0.88,0.93)). I: Regional association plot for rs117715768 (OR=1.15, 95% CI = (1.09,1.21)). J: Regional association for rs1373058 (OR=1.07, 95% CI = (1.05,1.10)). K: Regional association for rs4268071 (OR=1.39, 95% CI = (1.25,1.54)). This variant has only discovery data.

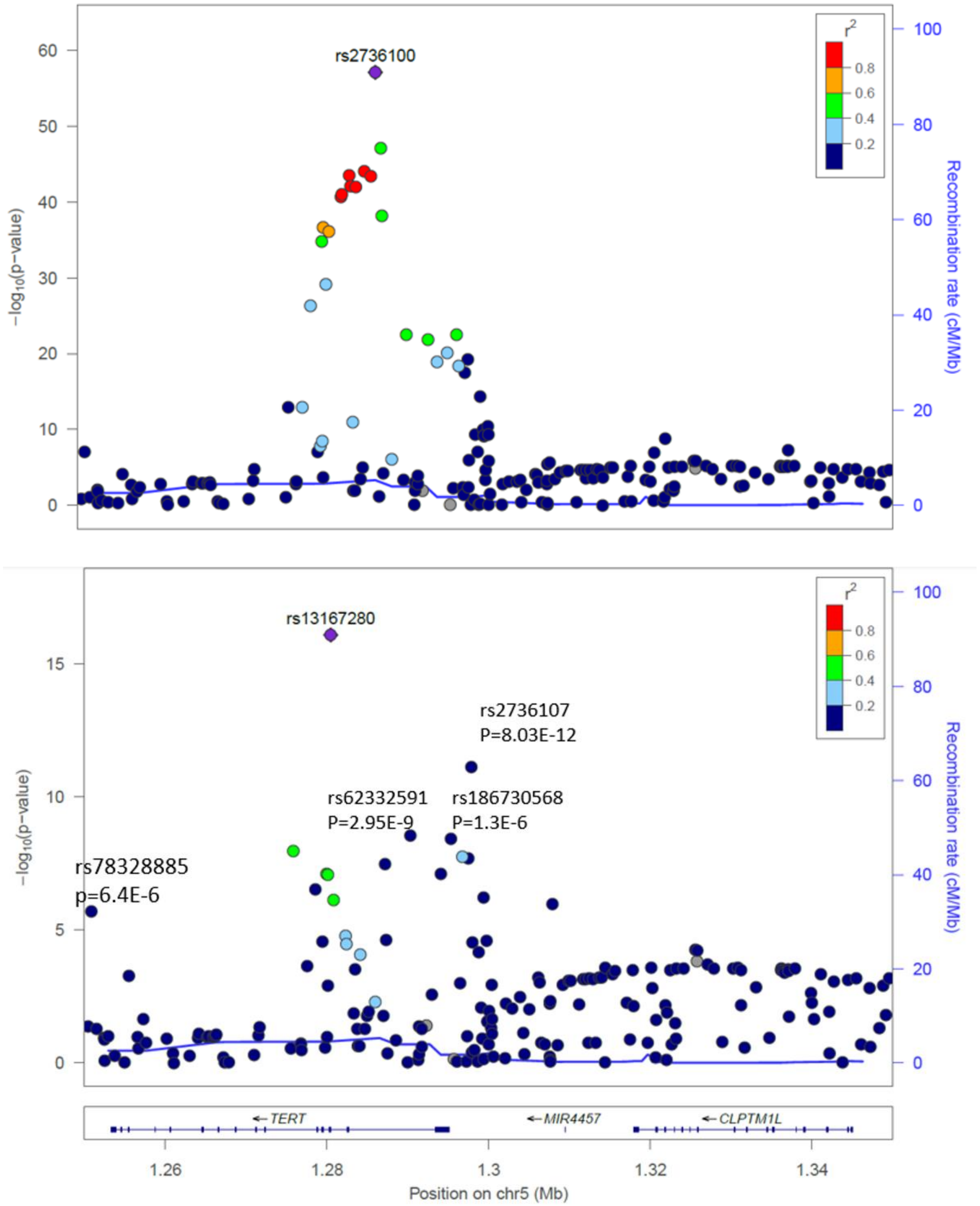

Supplementary Figure 2. Regional association plots of the *TERT-CLPTM1L* locus in EA populations. Top panel: Regional association plot with data from the GWAS meta-analysis. Bottom panel: Regional association plots with results conditioning on SNP rs2736100. All p-values were two-sided and not adjusted for multiple testing.

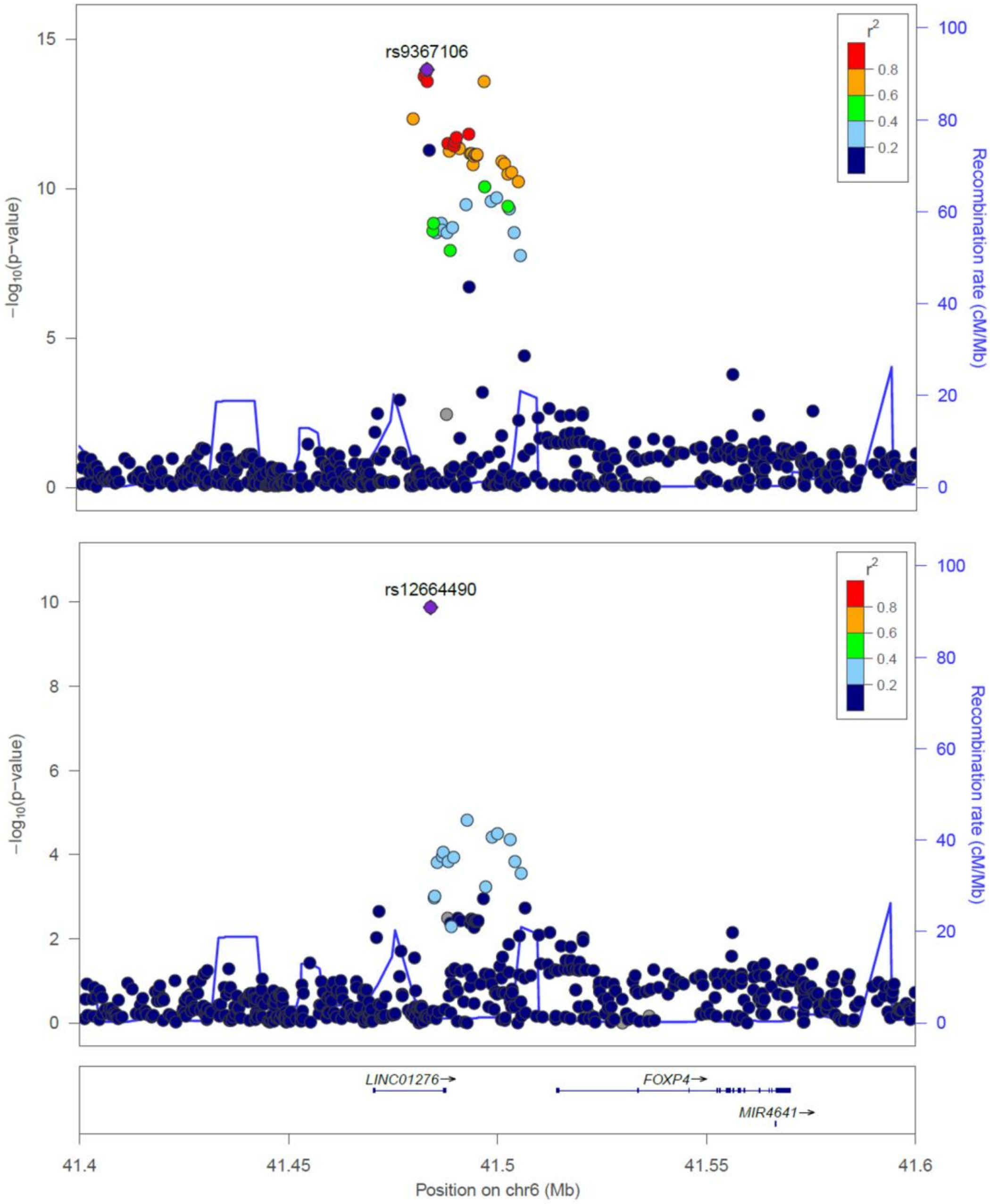

Supplementary Figure 3. Regional association plots of the *FOXP4* locus in EA populations. Top panel: Regional association plot with data from the GWAS meta-analysis. Bottom panel: Regional association plots with results conditioning on SNP rs9367106. All p-values were two-sided and not adjusted for multiple testing.

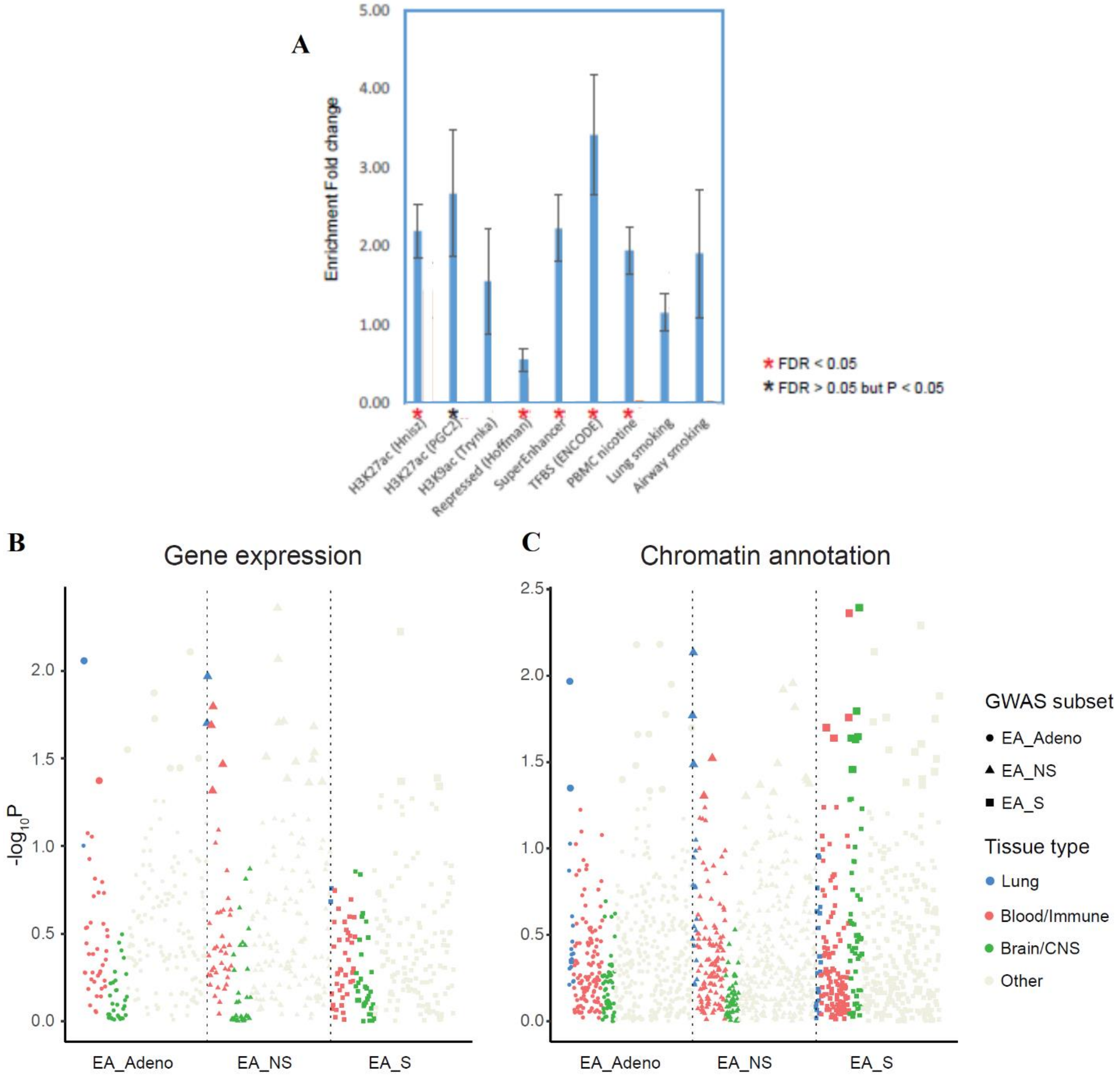

Supplementary Figure 4. Stratified LD score regressions (LDSC)[1] investigating functional enrichment and relevant tissues for LUAD heritability. (A) LUAD heritability enrichment in functional categories and smoking-relevant gene sets. Enrichment of heritability (center) and the 95% confidence intervals (error bar) are plotted for LUAD in each functional category. From 52 total categories including 24 main annotations and 500bp extension for each annotation, only those with significant enrichment (FDR<0.05) in EA are shown (full table in Supplementary Table 10). "PBMC nicotine" gene sets are from Moyerbrailean *et al.*[2], "Lung smoking" from Bosse et al.[3], and "Airway smoking" from Beane et al.[4]. (B-C) Stratified LDSC[1] of LUAD heritability for prioritizing relevant tissue types. Results are shown for tissue-specifically expressed genes from GTEx v6 (53 tissue types) and other public datasets (152 tissue types) (B) and for tissue-specific chromatin annotations from EnTEX (111 annotations in 26 tissue types) and Roadmap dataset (378 annotations in 85 tissue types) (C). $-\log_{10}$ (p) is plotted for each tissue type. Tissue types are grouped and color-coded for lung (blue), blood/immune (red), brain/CNS (green), or other (gray) for visualization. Tissue types with nominal P (one-sided) < 0.05 are shown in larger-size shapes.

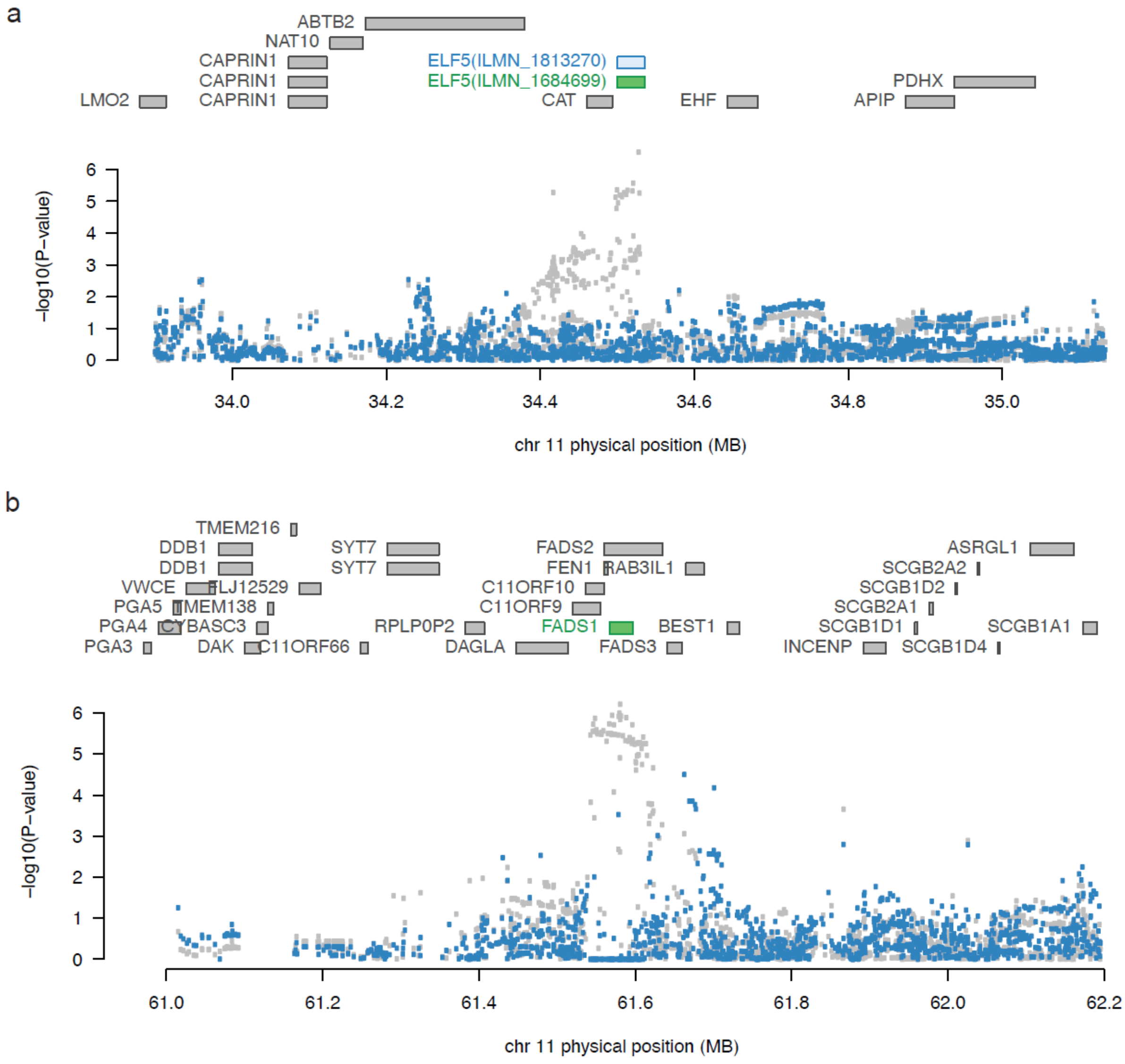

Supplementary Figure 5. Conditional analyses of significant TWAS loci using LCTCNS. The original LUAD GWAS p-values of the SNPs +/-500kb of the lead SNP are plotted in gray. GWAS p-values of the SNPs when conditioned on the predicted expression of *EFL5* (measured by the expression probe ILMN_1684699) (a) or *FADS1* (b) are shown in blue. The genes that are jointly TWAS significant are shown in green and those that are marginally significant are shown in blue.

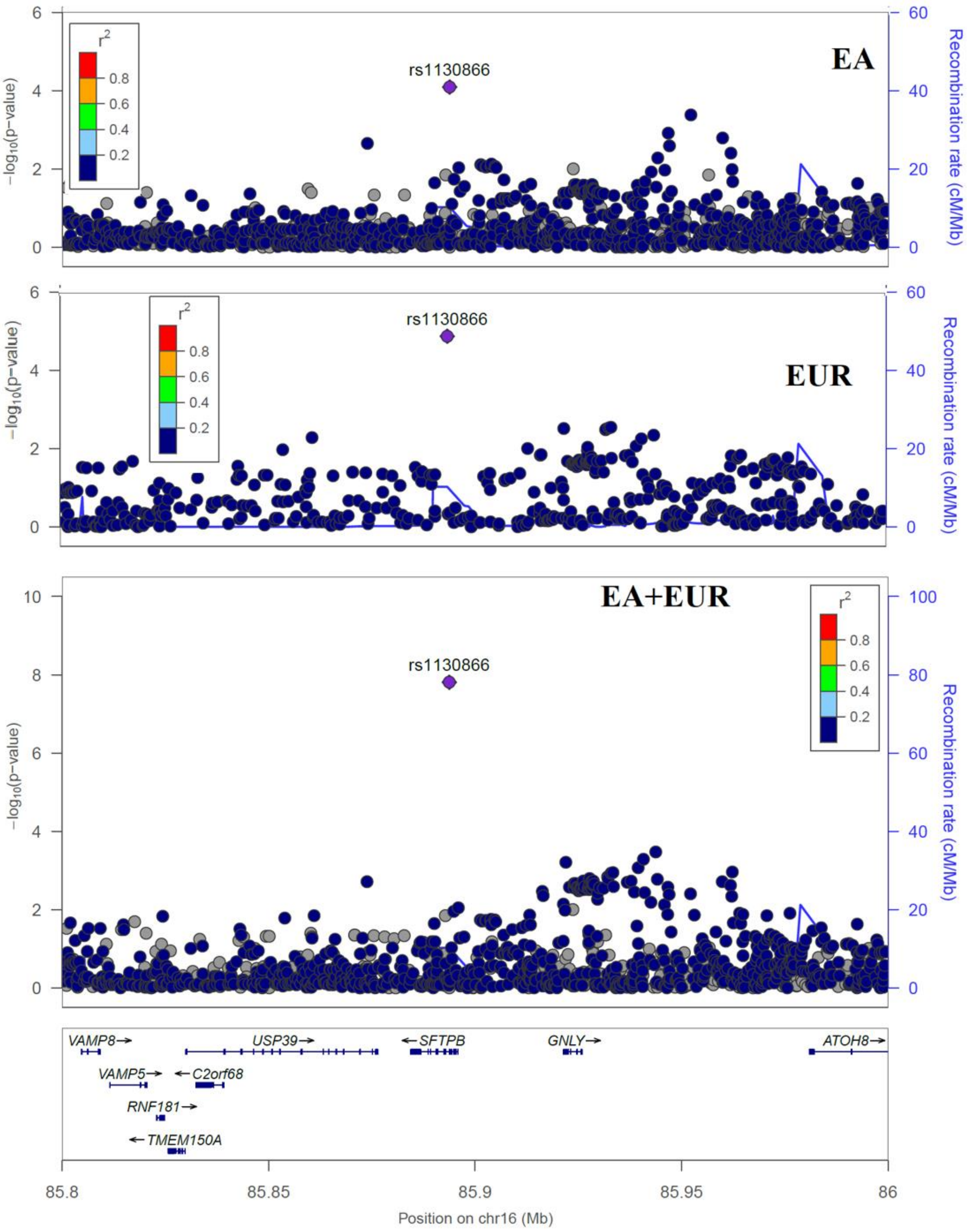

Supplementary Figure 6A: Regional association plots for rs1130866 identified based on multi-ancestry meta-analysis of East Asian (EA) and European (EUR) samples. The p-values were nominal and two-sided.

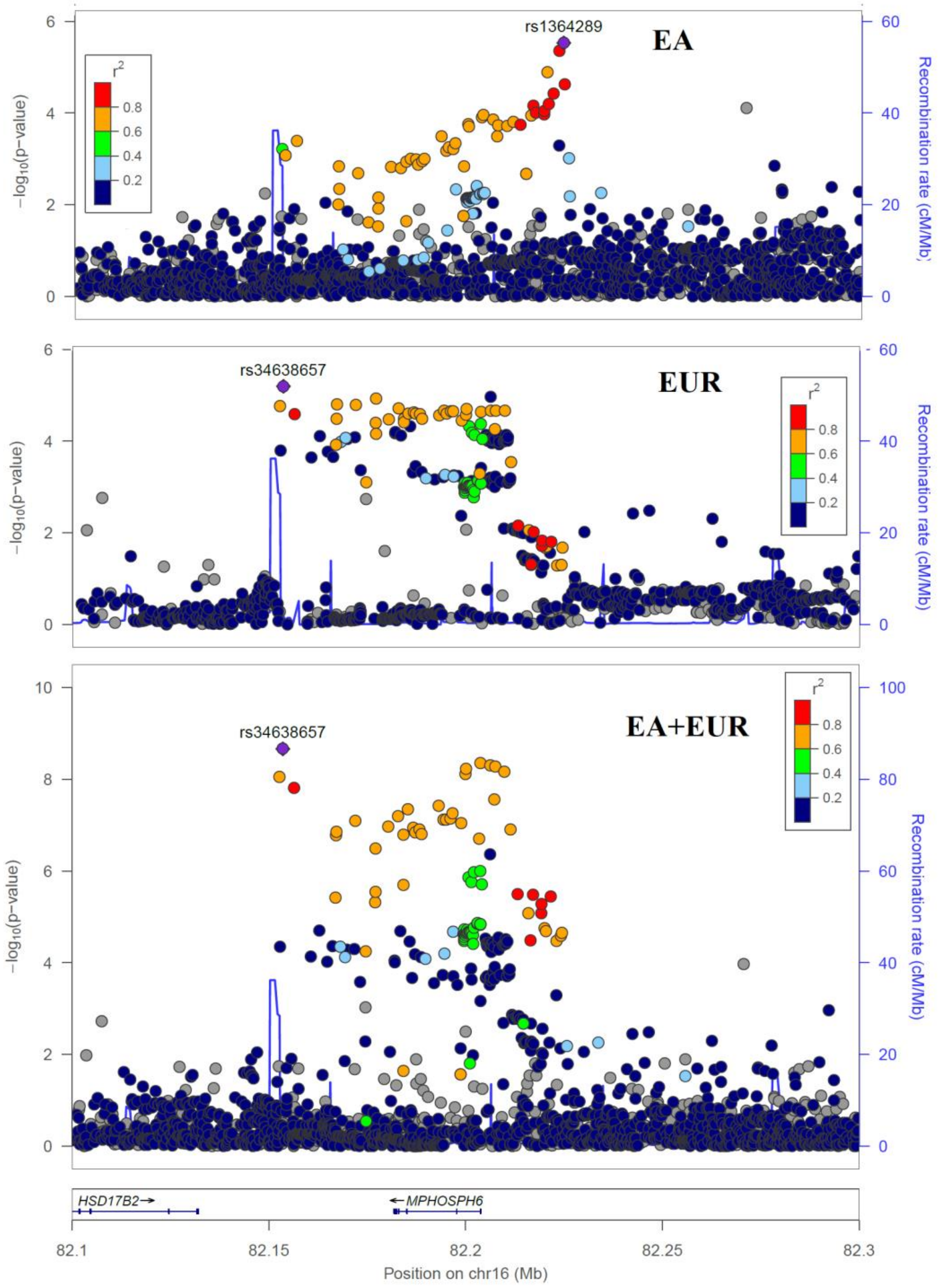

Supplementary Figure 6B: Regional association plots for rs34638657 identified based on multi-ancestry meta-analysis of EA and EUR samples. The p-values were nominal and two-sided.

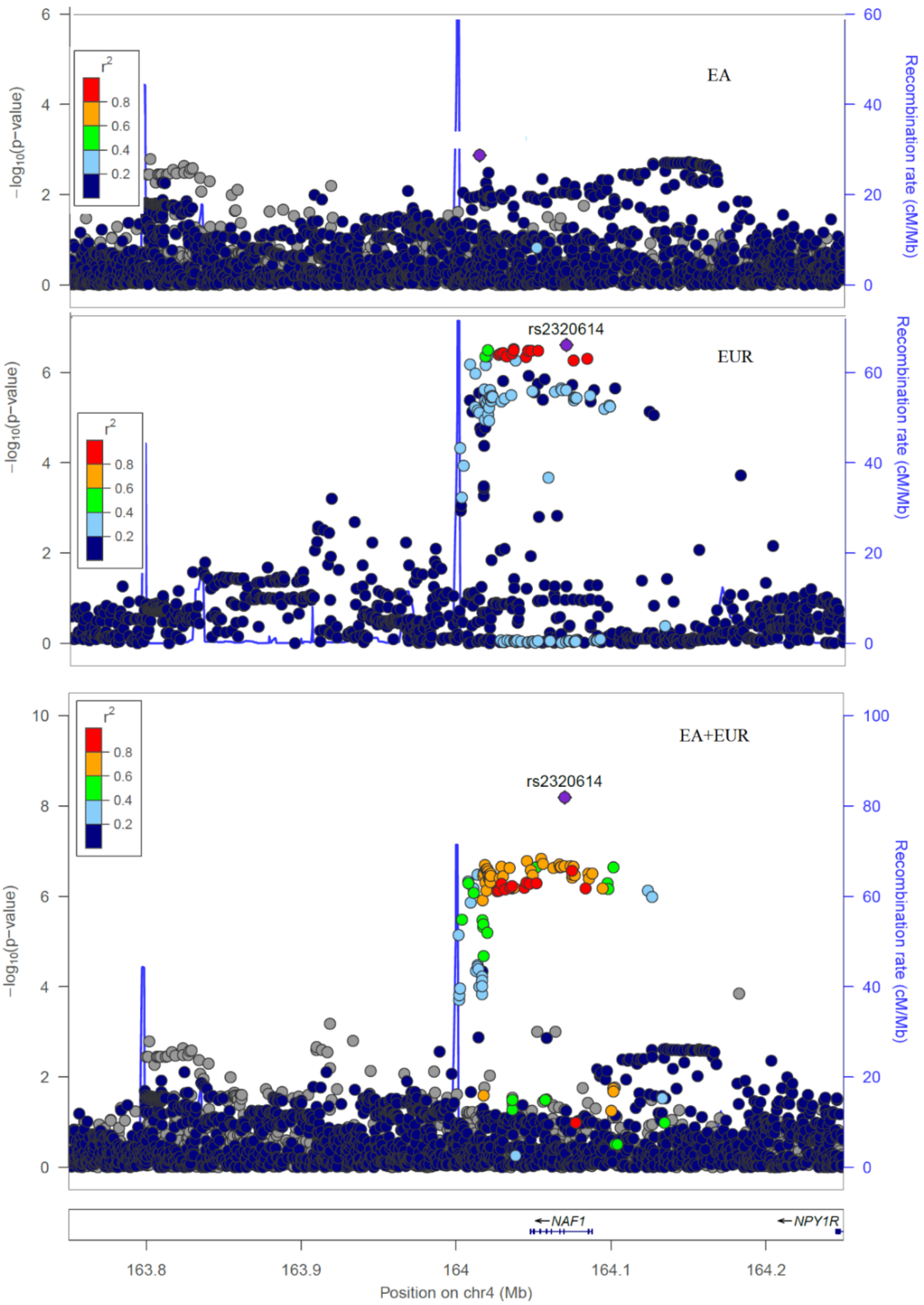

Supplementary Figure 6C: Regional association plots for rs2320614 identified based on multi-ancestry meta-analysis of EA and EUR samples. The p-values were nominal and two-sided.

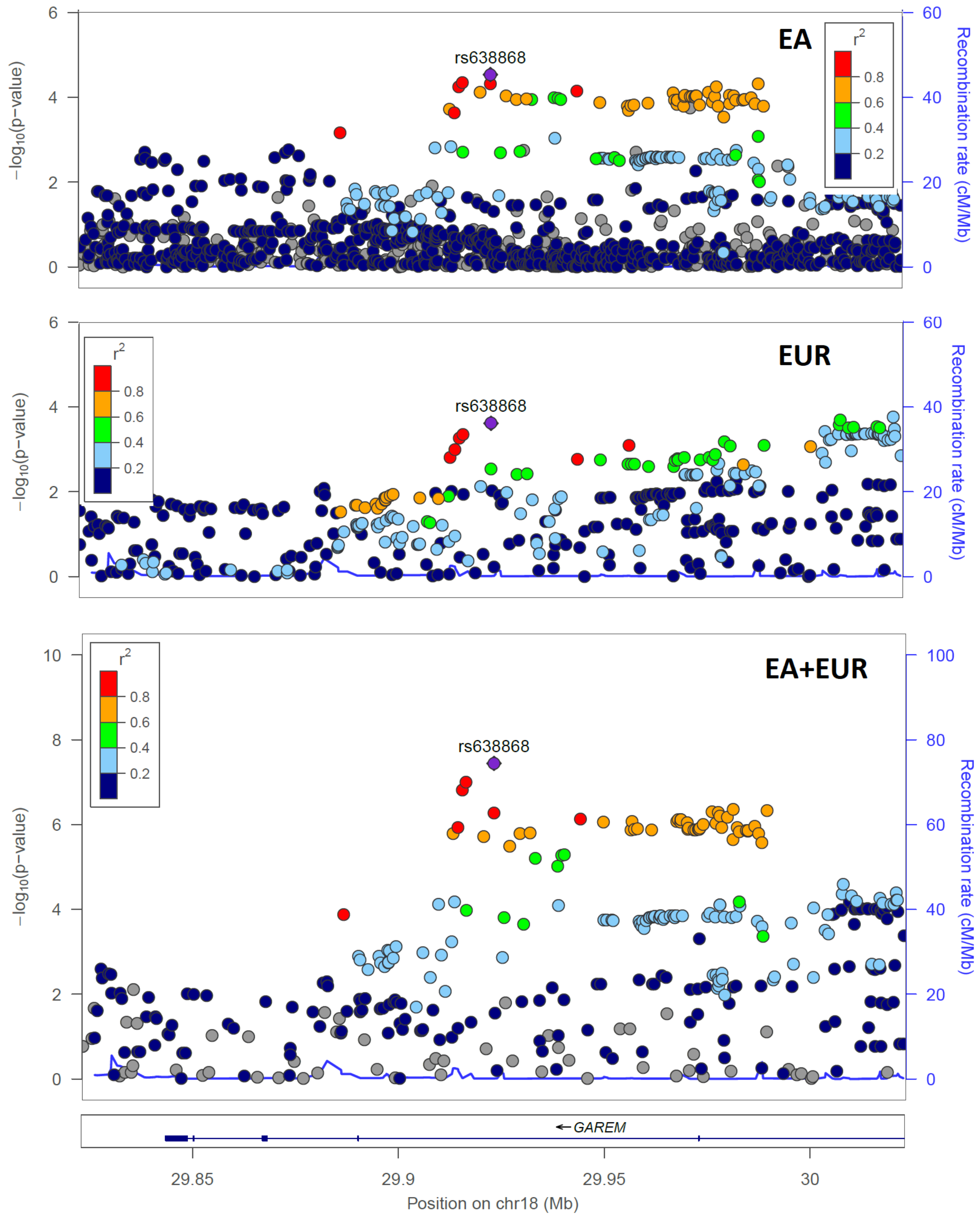

Supplementary Figure 6D: Regional association plots for rs638868 identified based on multi-ancestry meta-analysis of EA and EUR samples. The p-values were nominal and two-sided.

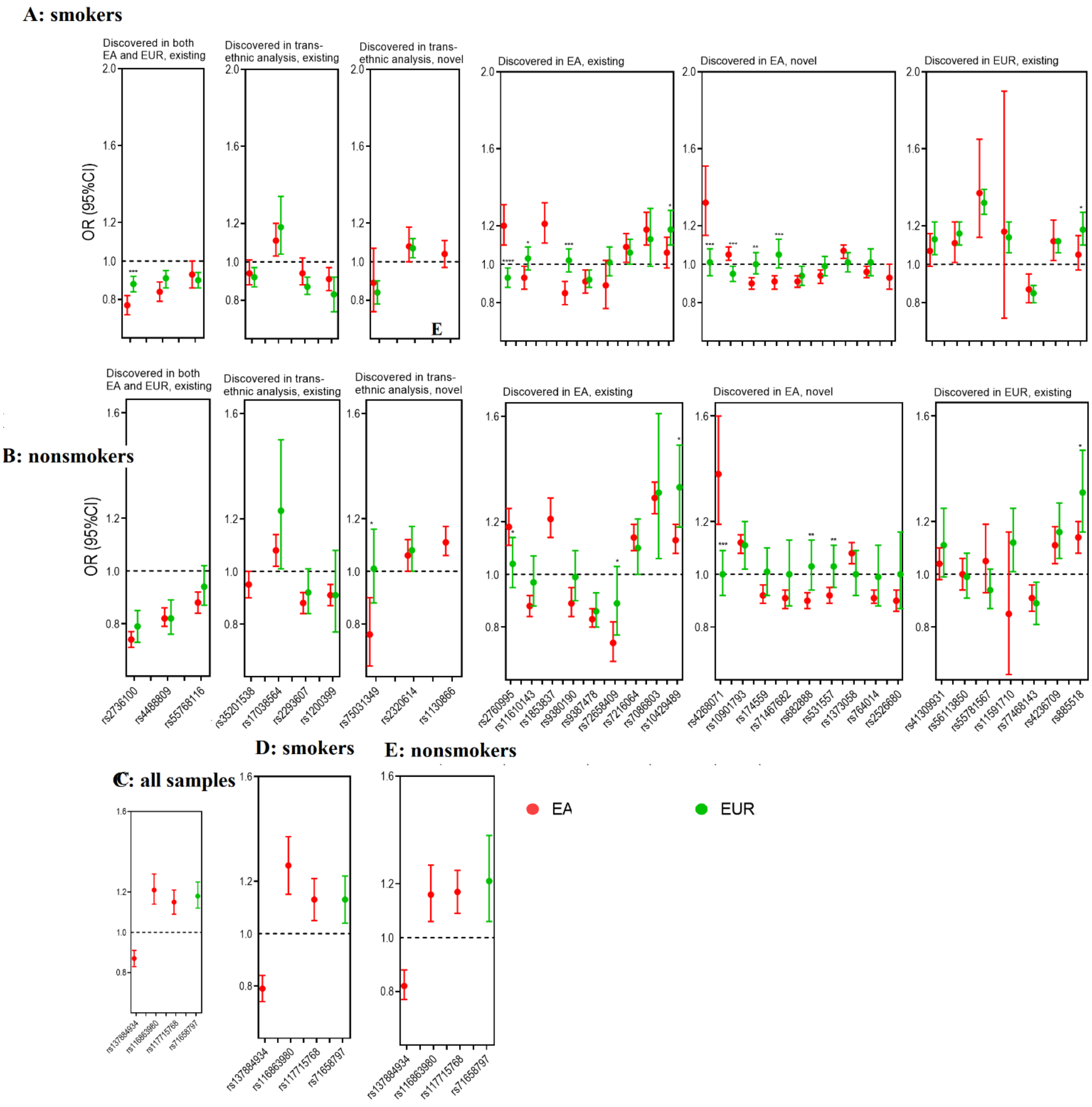

Supplementary Figure 7. Forest plots for lung adenocarcinoma susceptibility variants identified in East Asian (EA), European (EUR) or multi-ancestry analysis. A for smokers, B for non-smokers. C, D and E are for four SNPs with MAF <1% in either EA or EUR populations. SNPs are labeled with *, **, *** and **** correspond to $0.01 \leq p_{het} < 0.05$, $0.001 \leq p_{het} < 0.01$, $0.0001 \leq p_{het} < 0.001$ and $p_{het} < 0.0001$, respectively; here, p_{het} is the p-value for testing the heterogeneity of effect sizes between EA and EUR populations.

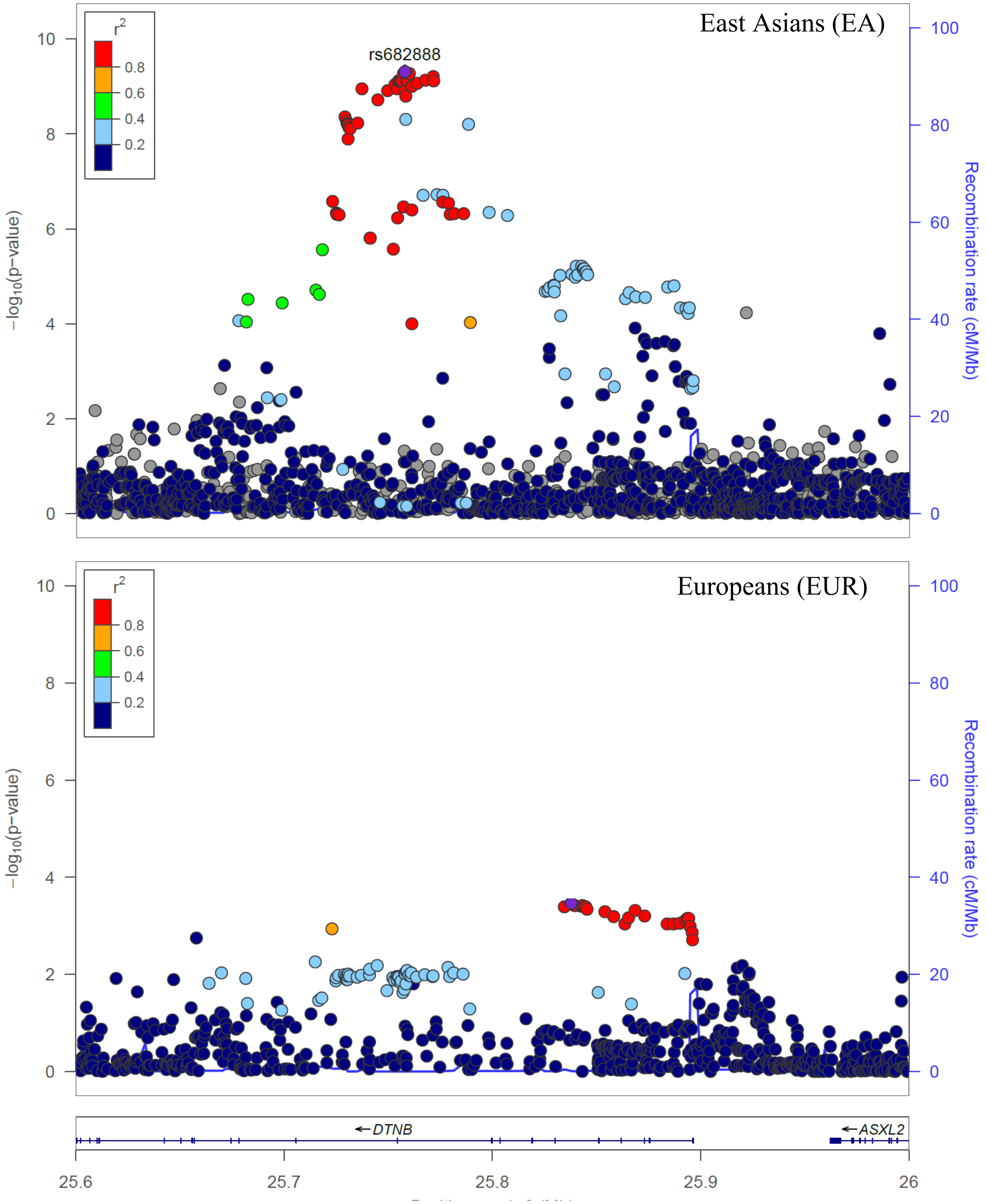

Supplementary Figure 8A. Regional association plots for rs682888, previously reported for East Asian (EA) populations, show no or very weak association in EUR populations. The p-values are nominal and two-sided.

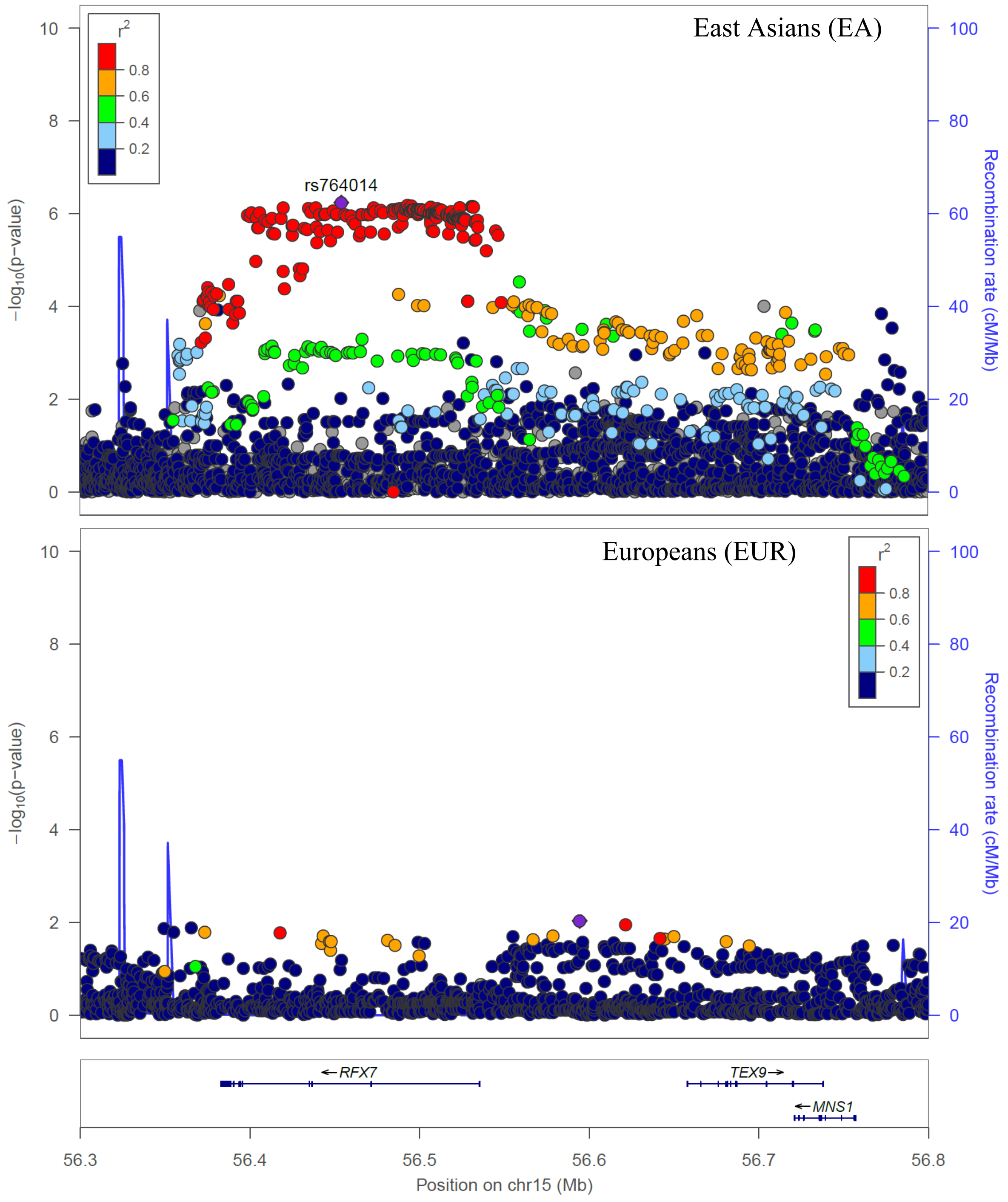

Supplementary Figure 8B. Regional association plots for rs764014, identified for EA populations, show no or very weak association in EUR populations. The p-values are nominal and two-sided.

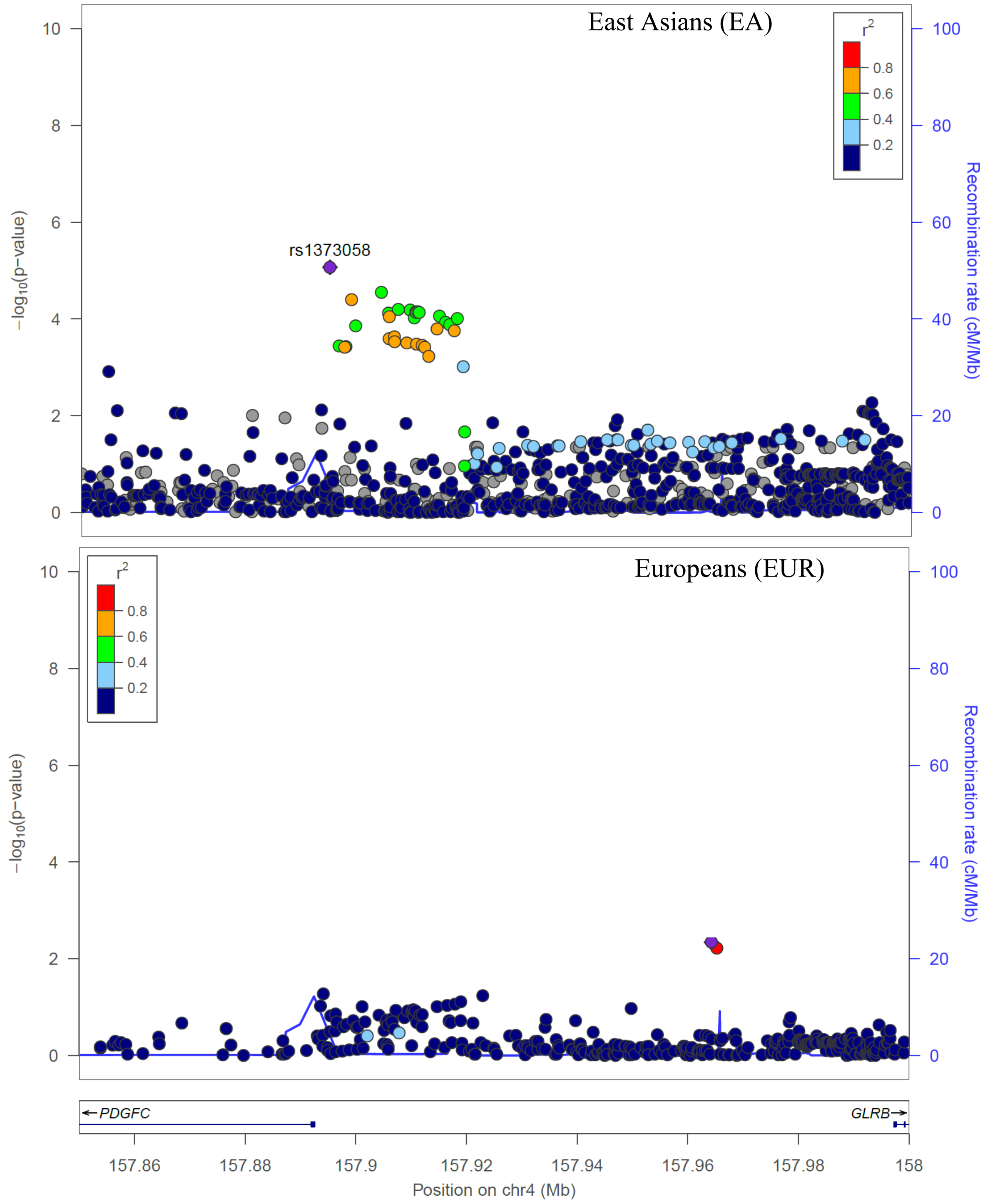

Supplementary Figure 8C. Regional association plots for rs1373058, previously reported for EA populations, show no or very weak association in EUR populations. The p-values are nominal and two-sided.

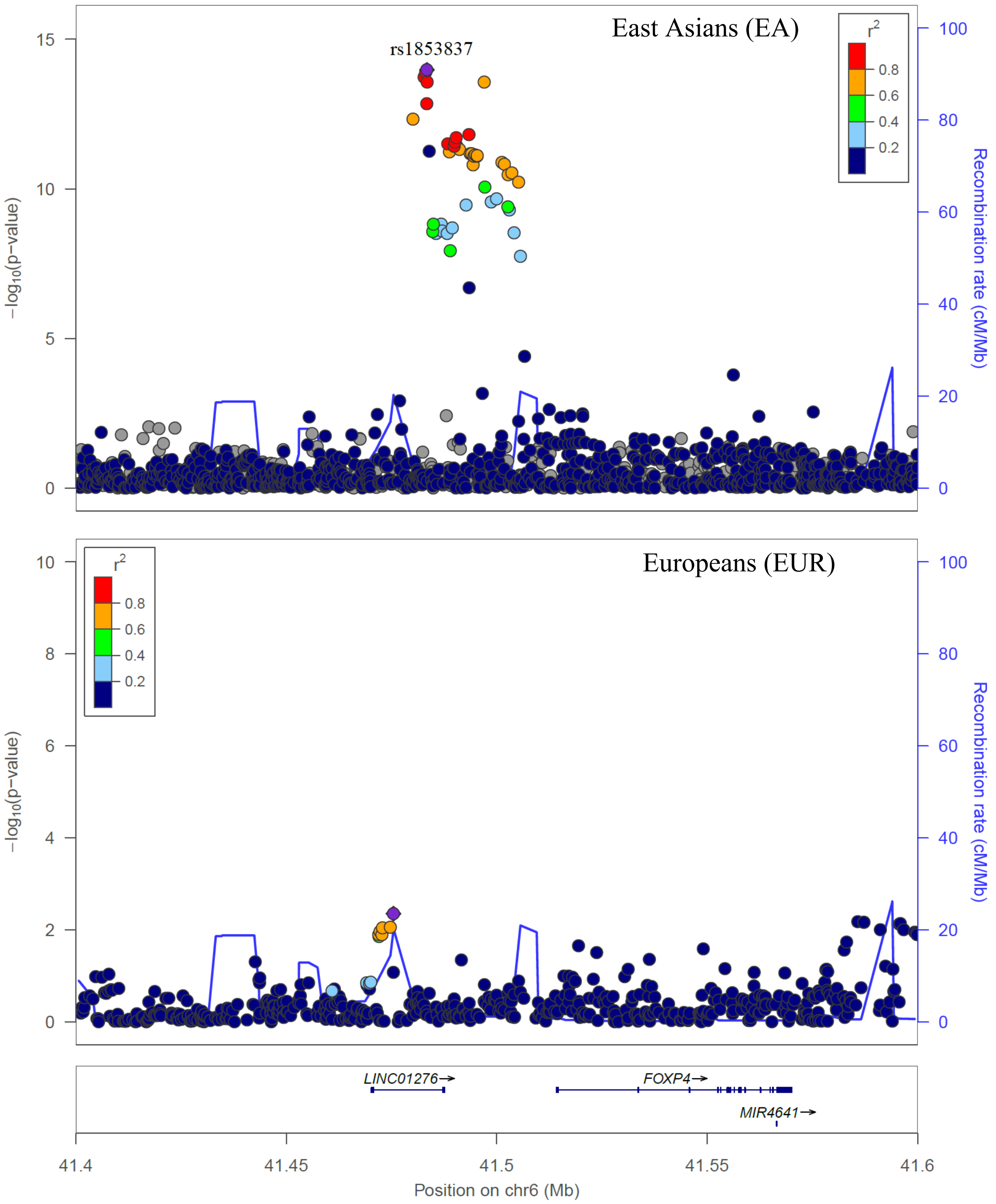

Supplementary Figure 8D. Regional association plots for rs1853837, previously reported for EA populations, show no or very weak association in EUR populations. The p-values are nominal and two-sided.

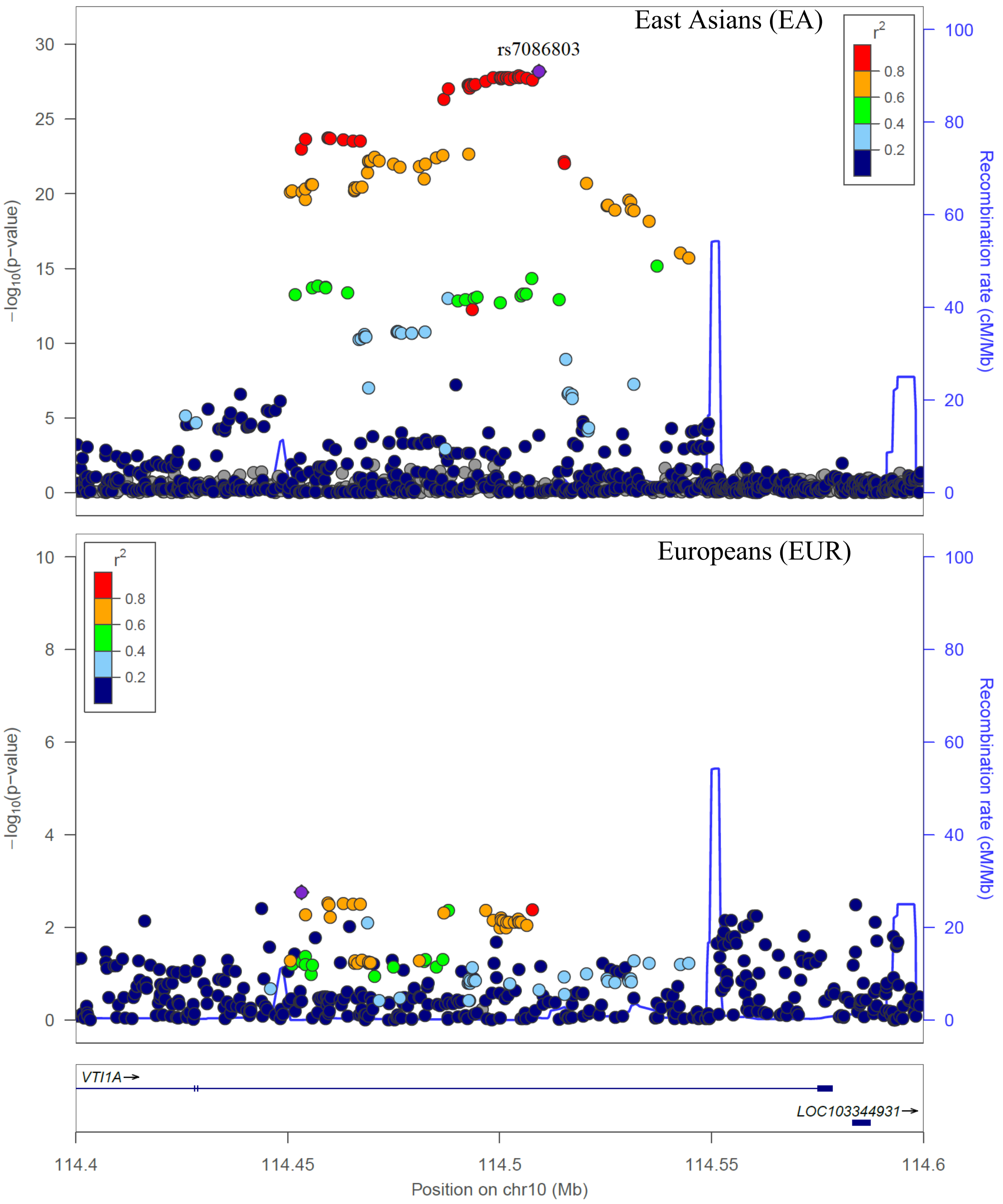

Supplementary Figure 8E. Regional association plots for rs7086803, previously reported for EA populations, show weak association in EUR populations. The p-values are nominal and two-sided.

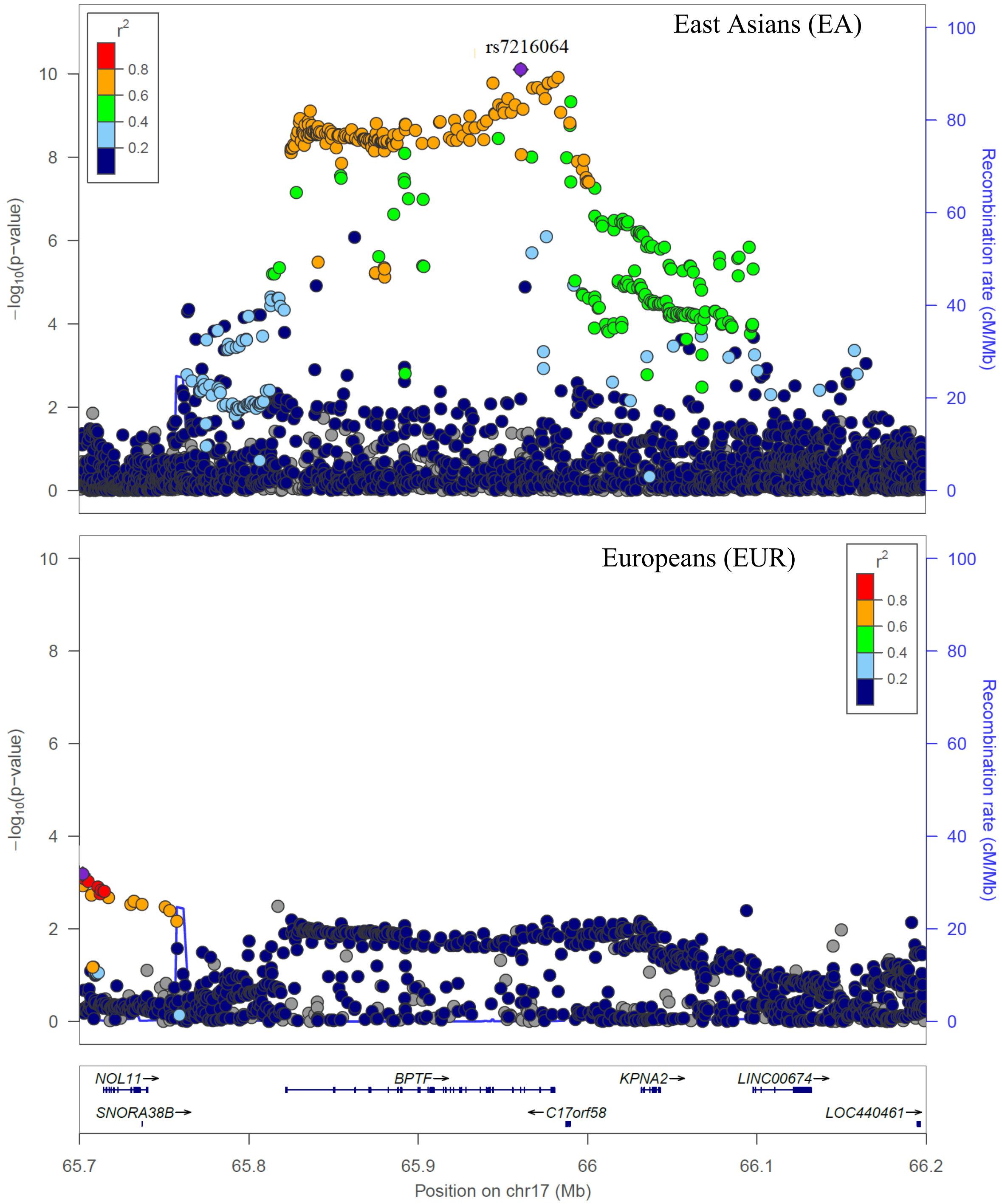

Supplementary Figure 8F. Regional association plots for rs7216064, previously reported for EA populations, show very weak association in EUR populations. The p-values are nominal and two-sided.

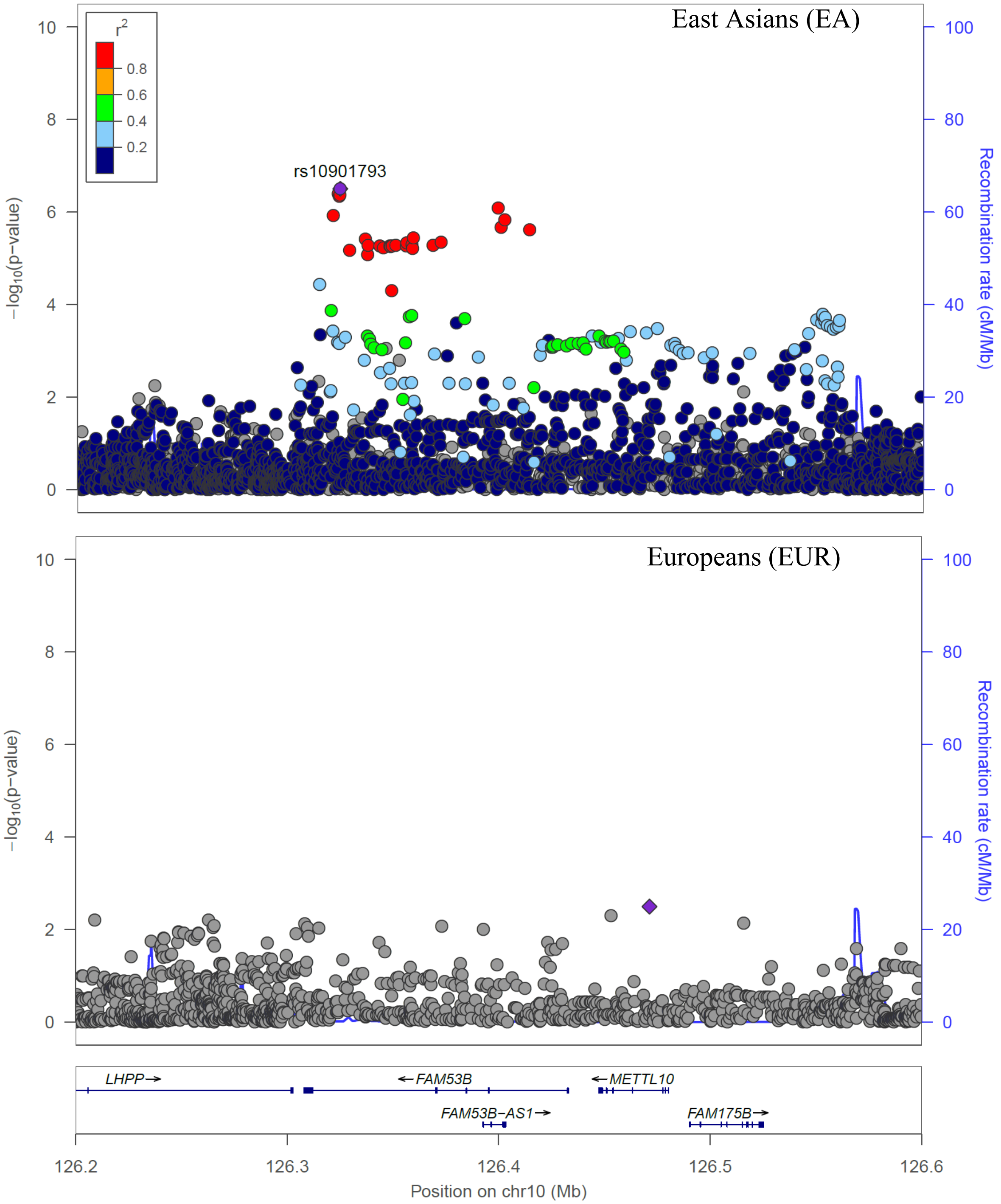

Supplementary Figure 8G. Regional association plots for rs10901793, previously reported for EA populations, show no association in EUR populations. The p-values are nominal and two-sided.

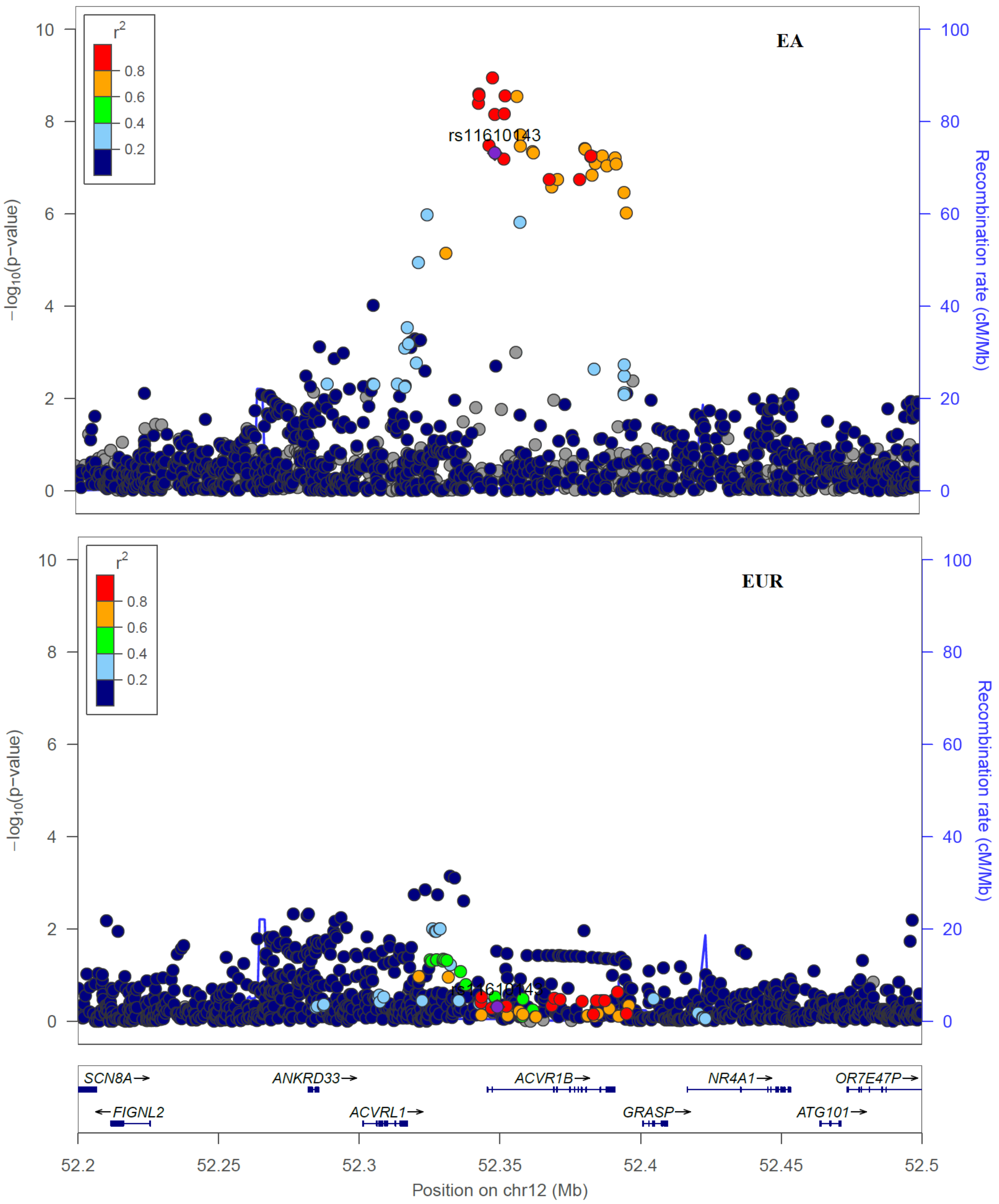

Supplementary Figure 8H. Regional association plots for rs11610143, previously reported for EA populations, show no association in EUR populations. The p-values are nominal and two-sided.

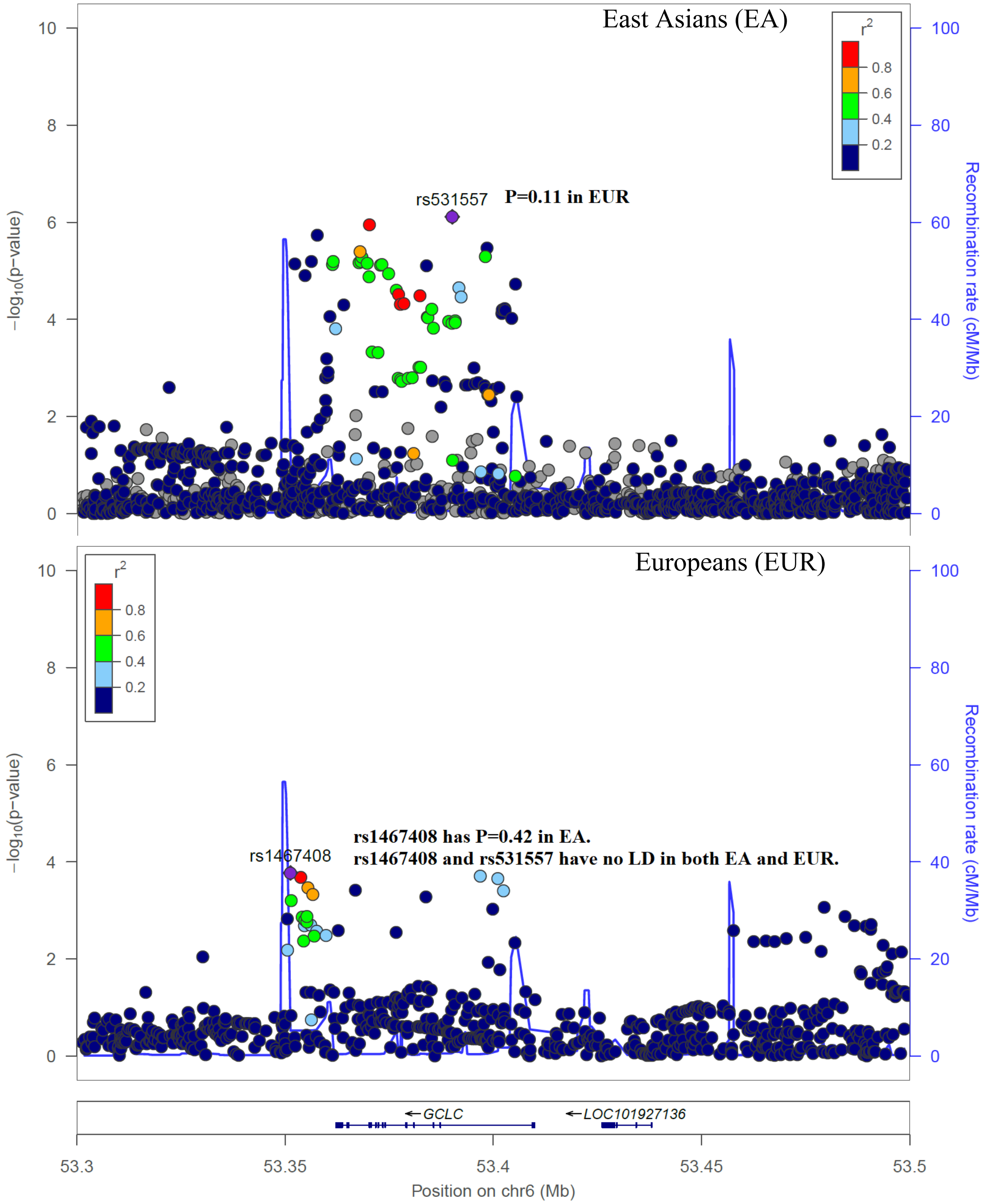

Supplementary Figure 8I. Regional association plots for rs531557 identified for EA populations. The SNP has no signal in EUR. An independent SNP rs1467408 showed modest association in EUR populations, which warrants further study in EUR populations. The p-values are nominal and two-sided.

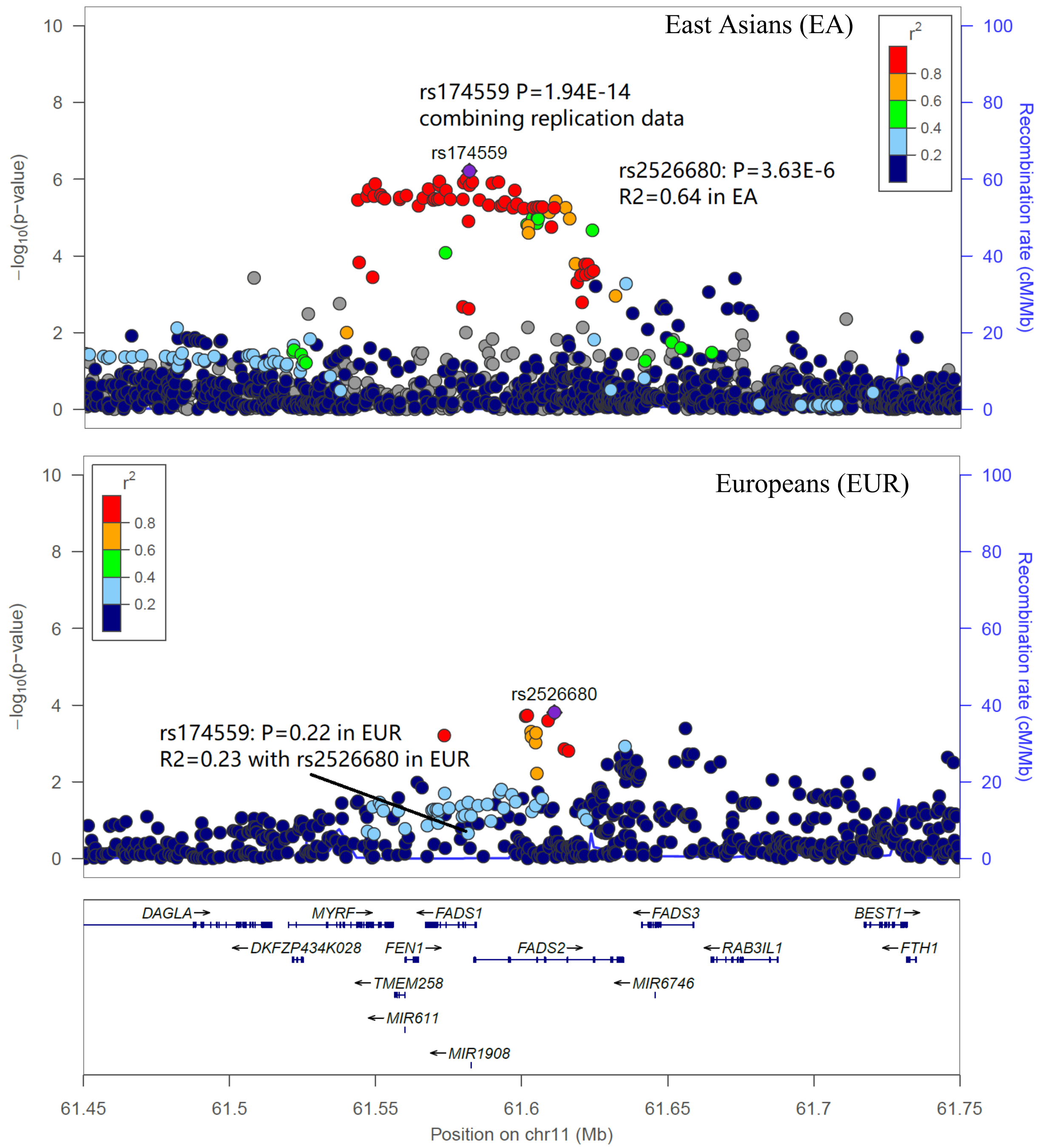

Supplementary Figure 8J. Regional association plots for rs74559 identified for EA populations. The SNP has no signal in EUR (p=0.22). Another SNP rs2526680 in modest LD with rs174559 showed modest association in EUR. Moreover, the trans-ethnic meta-analysis combing EA (discovery phase) and EUR showed that rs2526680 was the strongest SNP in the region. The p-values are nominal and two-sided.

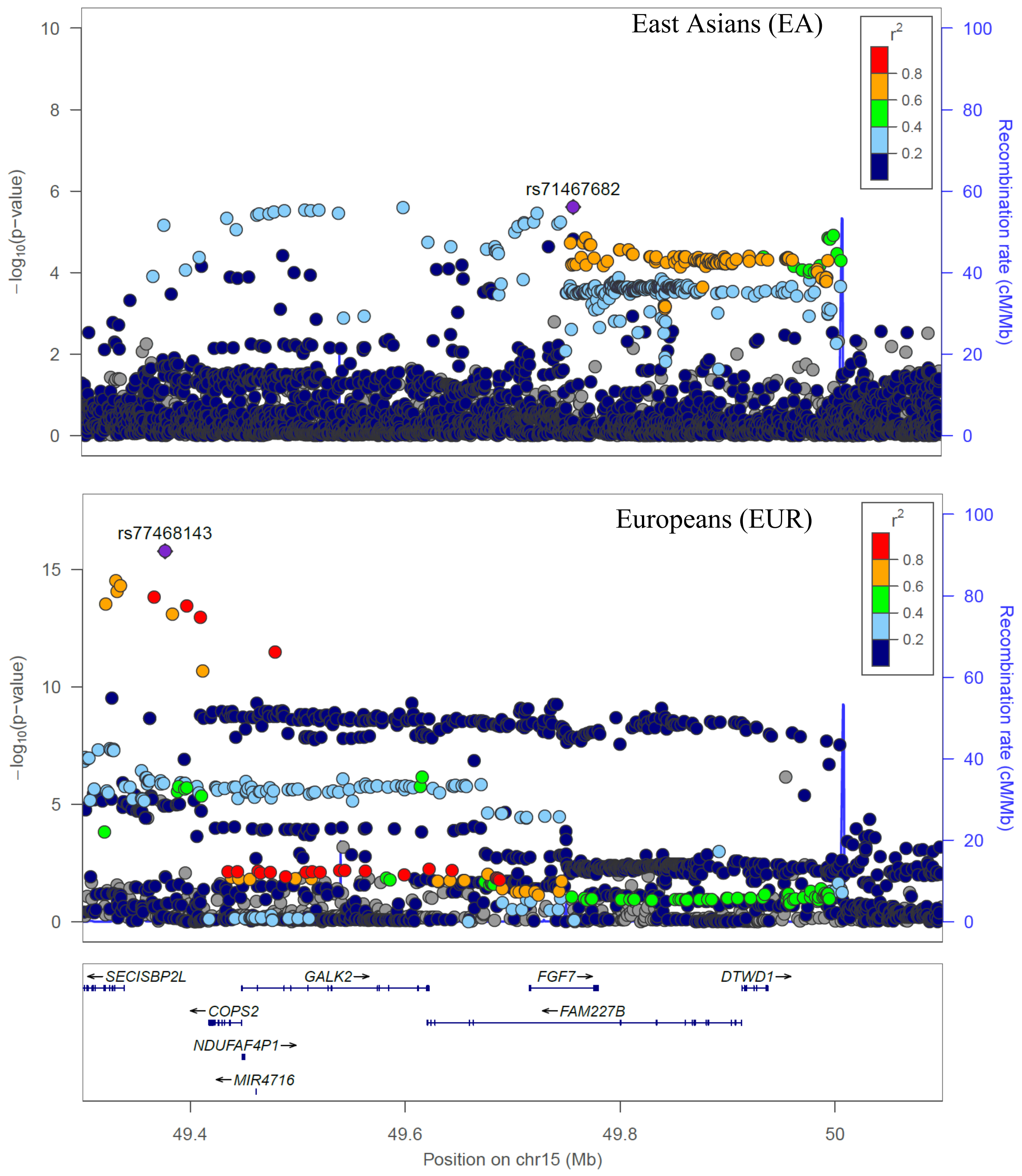

Supplementary Figure 8K. Regional association plots for rs71467682 reported for EA populations, and rs77468143 previously identified in EUR populations. The two SNPs have no LD in EUR and weak LD in EA. The p-values are nominal and two-sided.

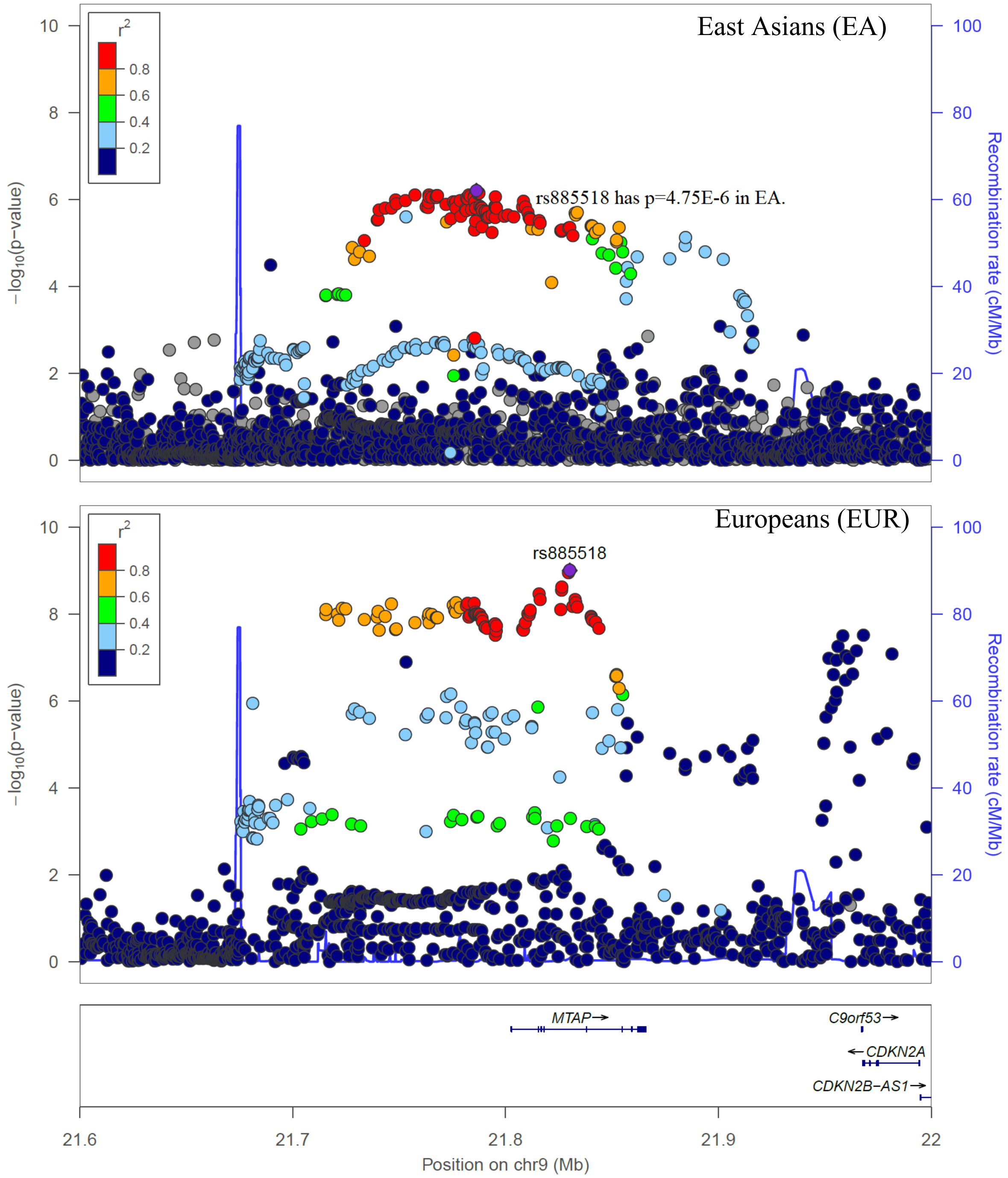

Supplementary Figure 9A. Regional association plots for rs885518, previously reported for European (EUR) populations also show association in East Asian (EA) populations. The p-values are nominal and two-sided.

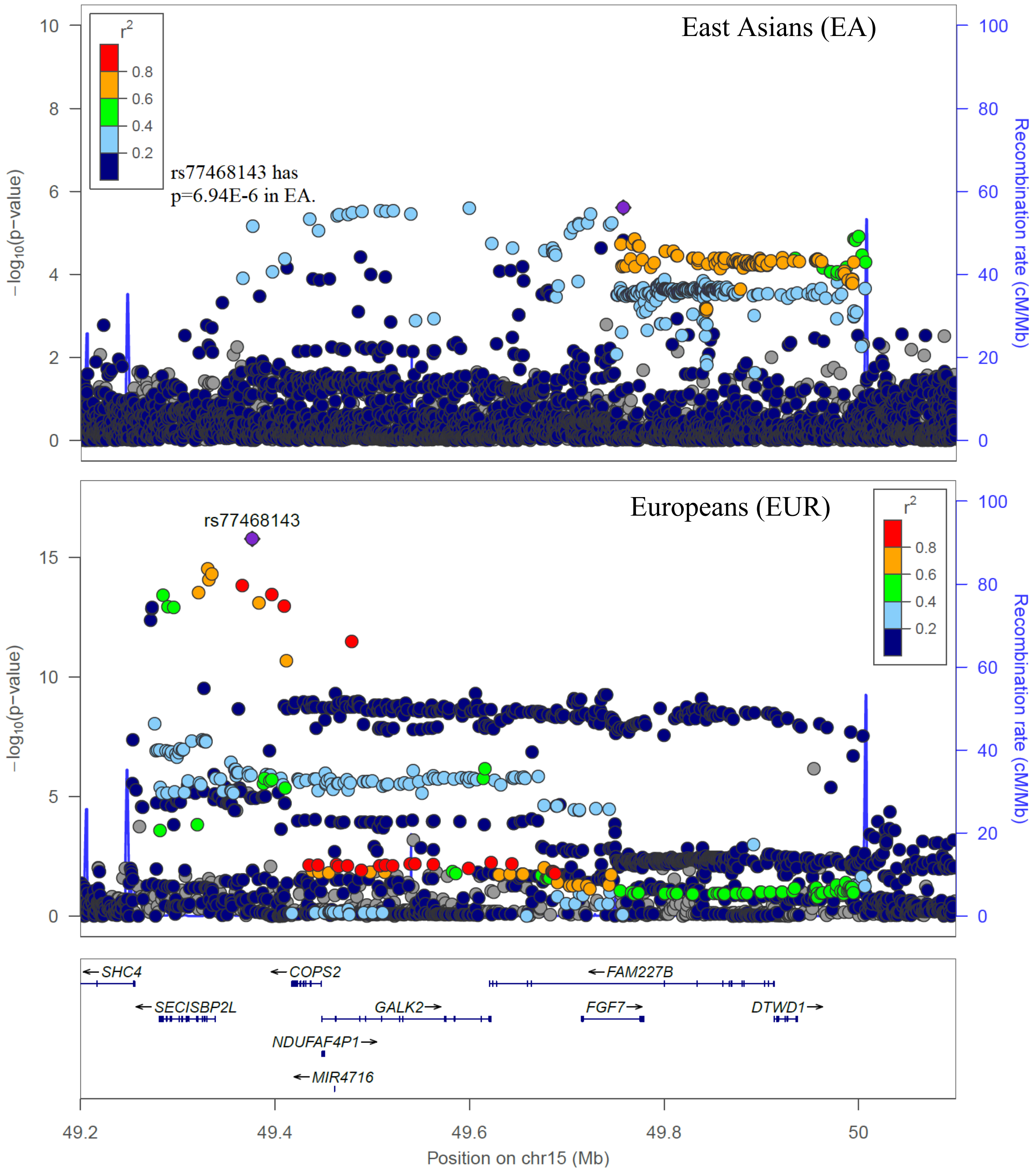

Supplementary Figure 9B. Regional association plots for rs77468143, previously reported for EUR populations also show association in EA populations. The p-values are nominal and two-sided.

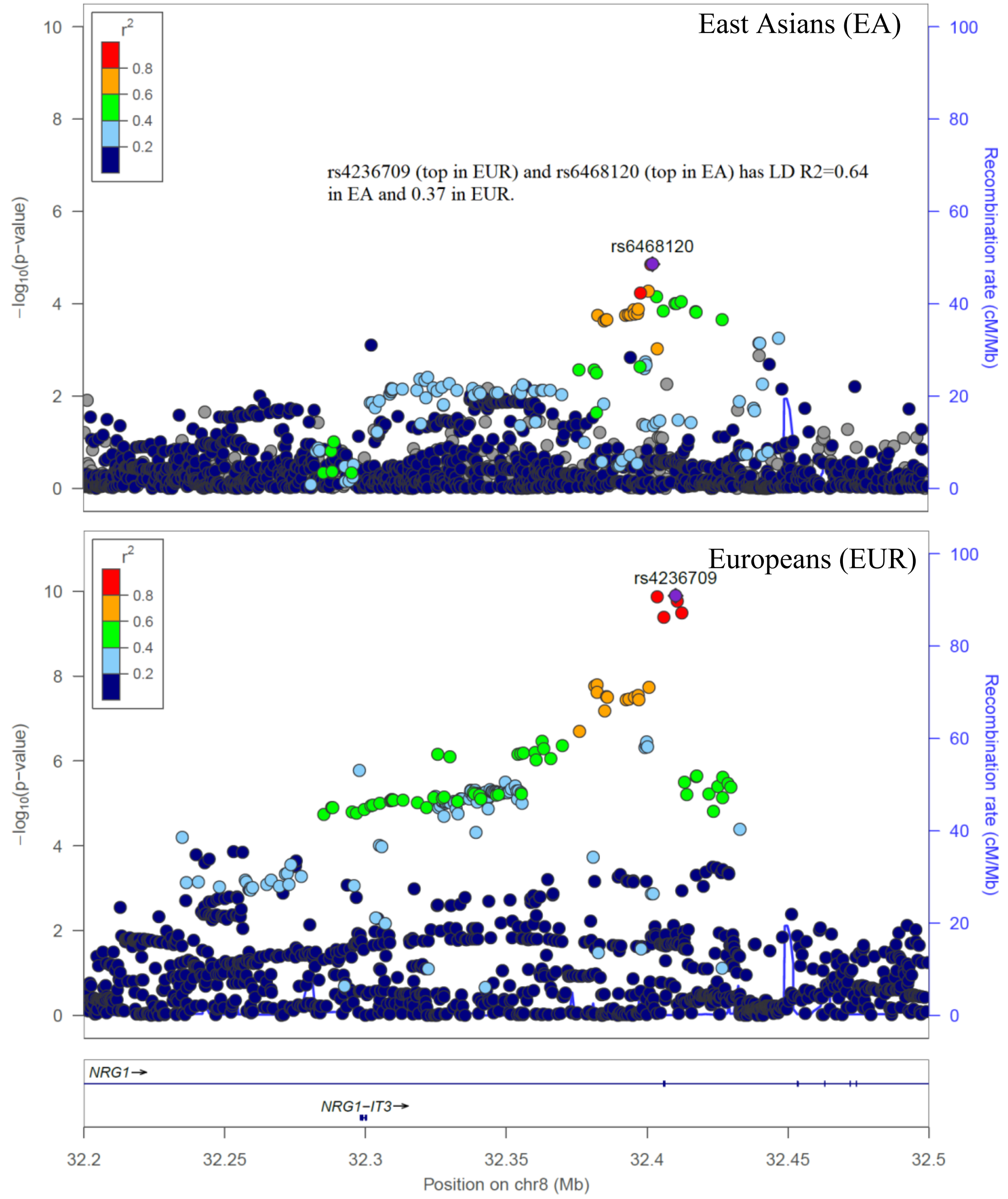

Supplementary Figure 9C. Regional association plots for rs4236709, previously reported for European populations also show association in EA populations. Rs4236709 has p=9.81E-5 in EA; the top SNP in EA rs6468120 has a slightly stronger association. The p-values are nominal and two-sided.

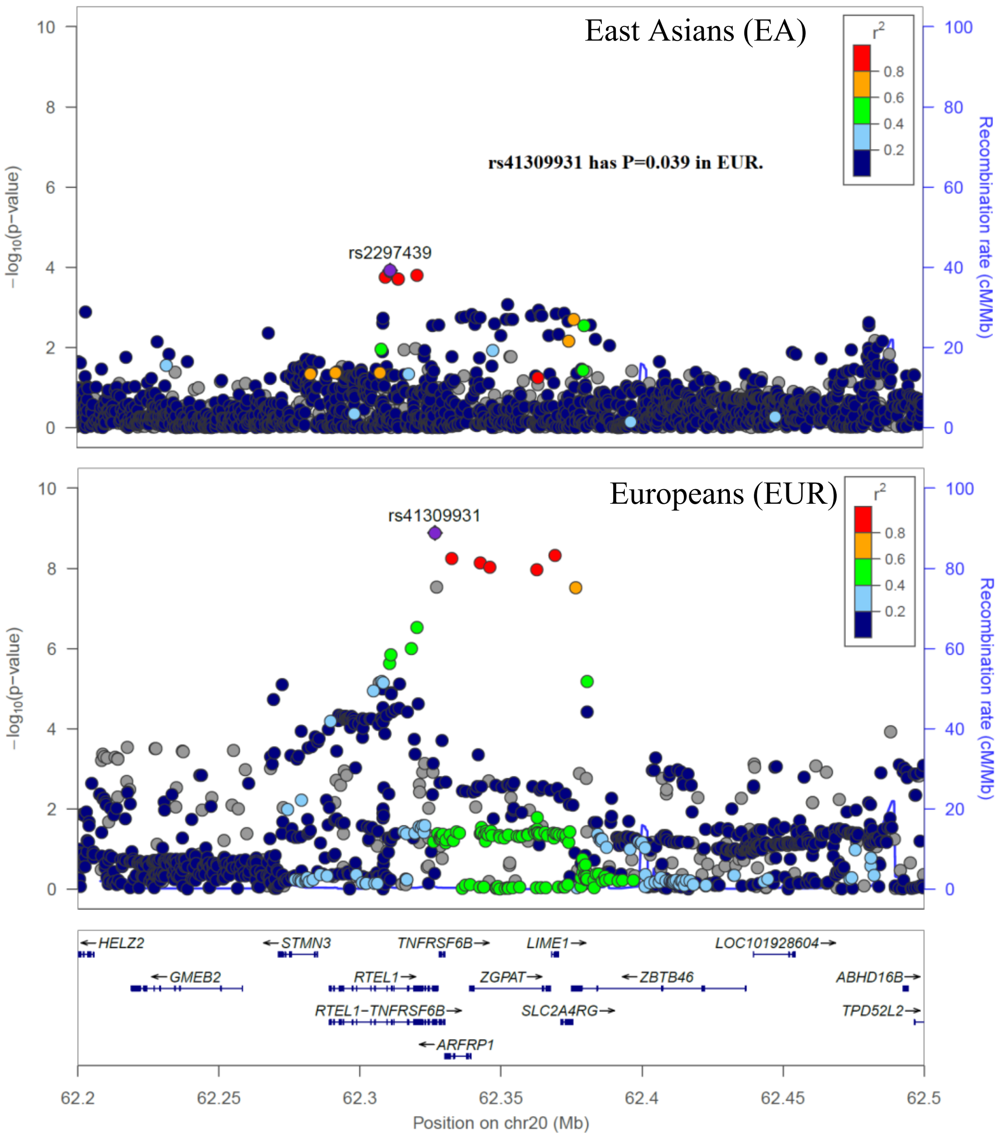

Supplementary Figure 9D. Regional association plots for rs41309931, previously reported for European populations show weak association in EA populations.

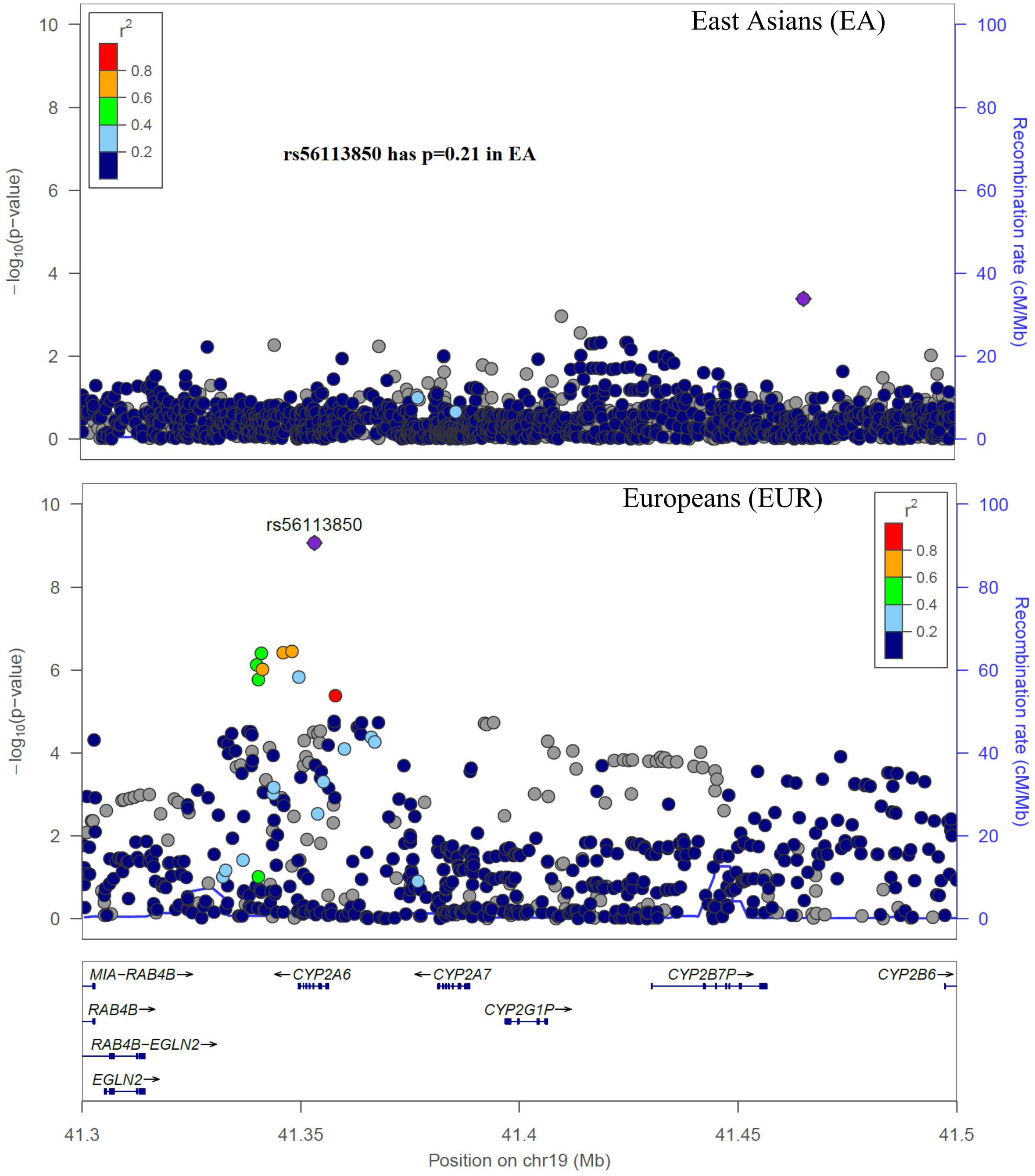

Supplementary Figure 9E. Regional association plots for rs56113850, previously reported for European populations show no association in EA populations. The p-values are nominal and two-sided.

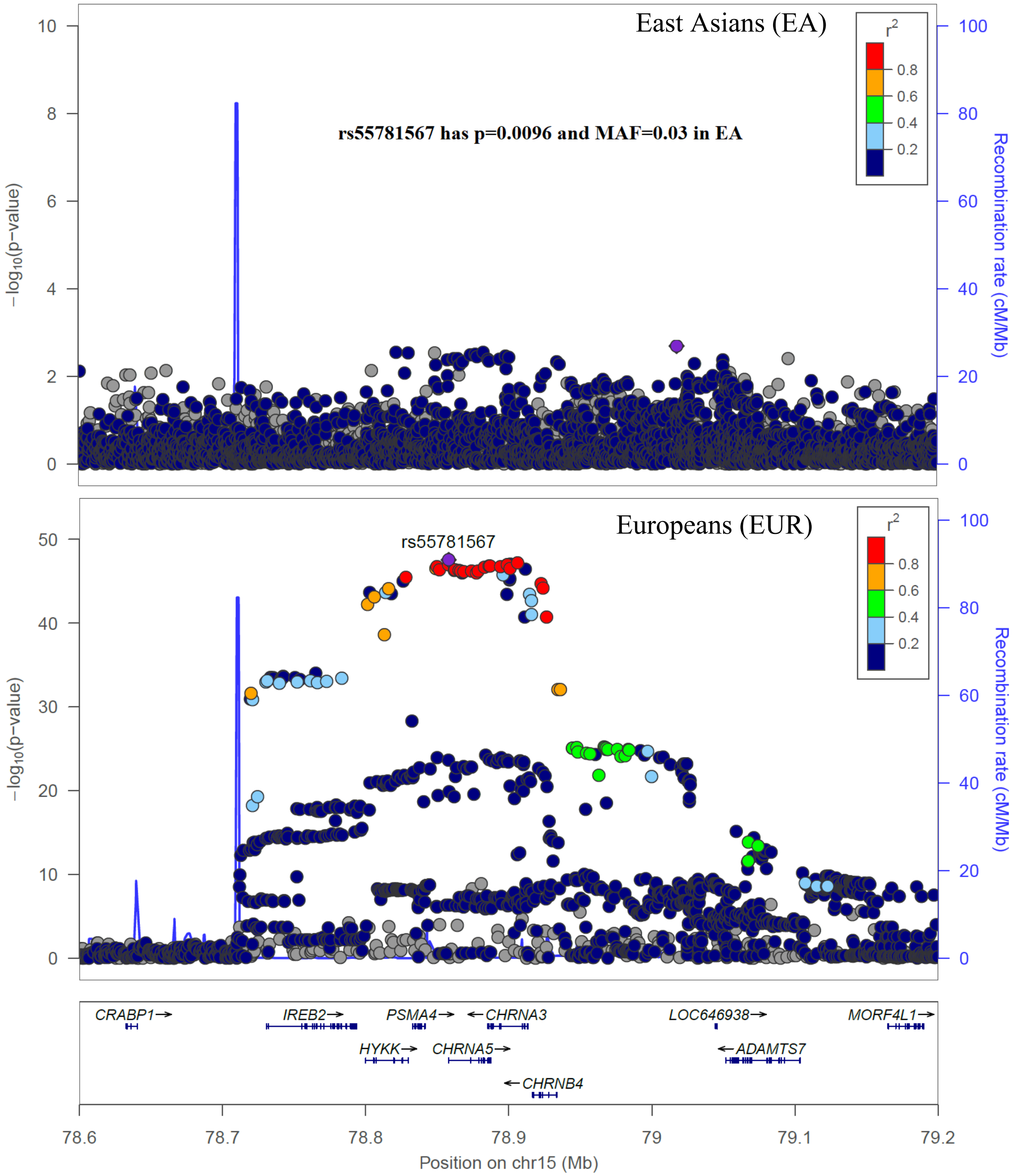

Supplementary Figure 9F. Regional association plots for rs55781567, previously reported for European populations show weak association in EA populations because of low MAF (0.03) in EA. The p-values are nominal and two-sided.

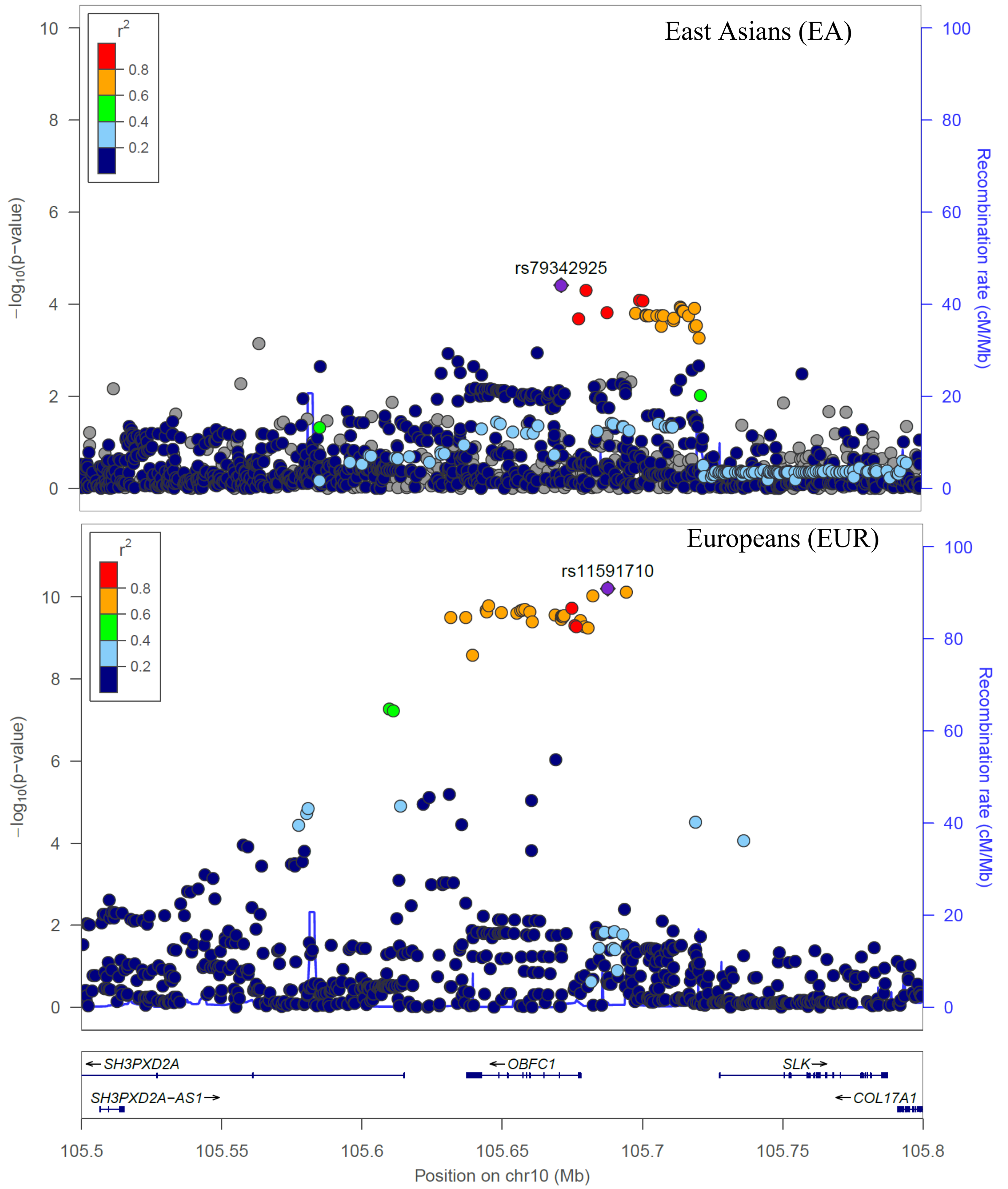

Supplementary Figure 9G. Regional association plots for rs11591710, previously reported for European populations. Rs11591710 shows no association in EA populations with p=0.64 in EA. In the same region, an independent SNP rs79342925 has p=6.5E-5 in EA; however, this SNP does not exist in EUR. The p-values are nominal and two-sided.

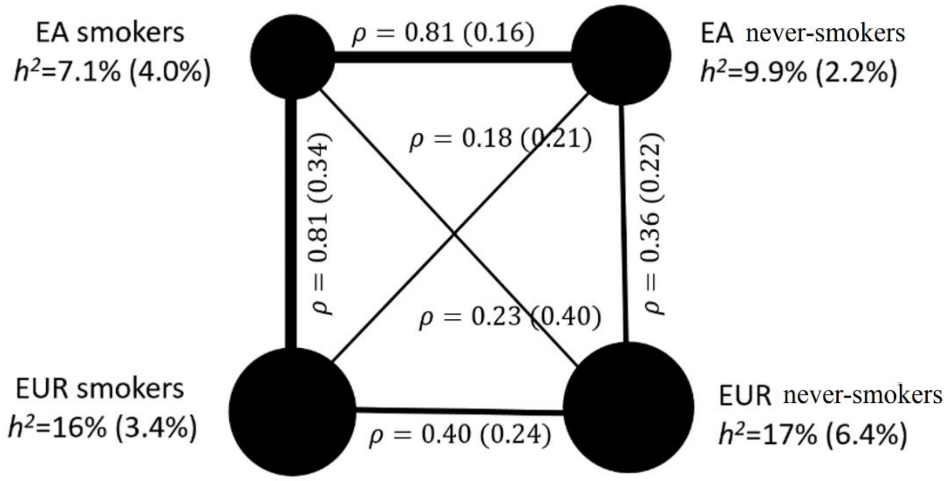

Supplementary Figure 10. Estimated heritability of lung adenocarcinoma and the genetic correlation coefficient between smokers and never-smokers using LDSC[4], between East Asian (EA) and European (EUR) populations using POPCORN. The sizes of the circles reflect the estimated heritability, and the thickness of lines reflects the estimated genetic correlations. The numbers in the parenthesis are standard error (s.e.). The heritability estimate is higher in EUR than in EA for both smokers and never-smokers; however, the differences are not statistically significant ($p_{het} = 0.09$ for smokers and p_{het} =0.23 for never-smokers). Moreover, rg between smokers and never-smokers was estimated to be higher in EA (rg=0.81, s.e.=0.16) than in EUR (rg=0.40, s.e.=0.24) but the difference is not statistically significant. We used POPCORN[5] to estimate trans-ethnic genetic-impact correlation between EA and EUR. We estimated that rg $=$ 0.81 (s.e.=0.34, p=0.29 for testing rg $= 1$) for smokers and rg $= 0.36$ (s.e.=0.22, p=0.002 for testing rg $= 1$) for never-smokers. To account for the difference of MAFs in two populations, we also used POPCORN to estimate the trans-ethnic genetic-impact correlation that was defined as the correlation of population specific phenotypic variance explained by each SNP. Results were consistent with the genetic correlation analysis with rg $= 0.79$ (s.e.=0.32) for smokers and rg $=$ 0.35 (s.e.=0.21) for never-smokers.

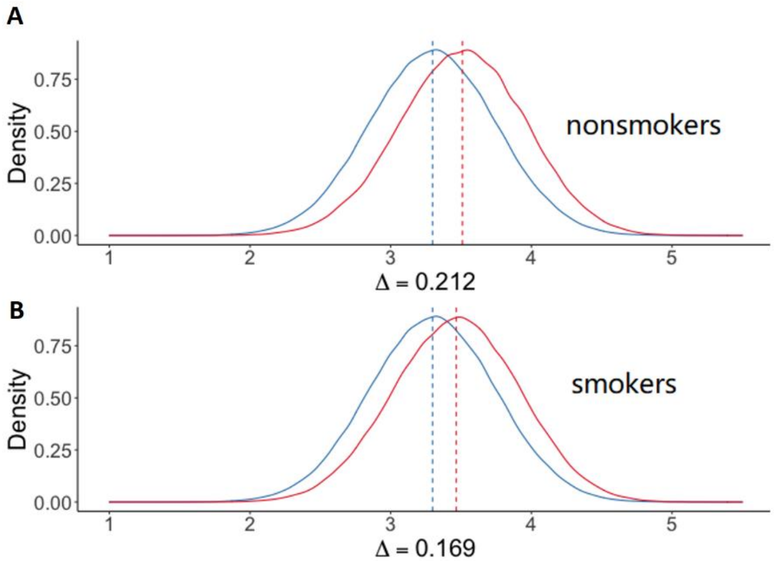

Supplementary Figure 11. Testing interaction between smoking status and polygenic risk scores (PRS). A. Distribution of PRS for never-smoking cases (red) and never-smoking controls (blue), calculated based on simulations with 25 variants in Supplementary Table 4. The weights of PRS are log(OR) with OR specified in Supplementary Table 4. EAF in controls are from Supplementary Table 4 and EAF for cases were calculated as $p * EAF/(1 - EAF + EAF * OR)$, where OR was based on never-smokers in the table. Δ measures the difference of average PRS values between cases and controls; a larger value of Δ indicates a stronger association between PRS and disease risk. B. Distributions of simulated PRS for smokers. The two Δ values (0.212 and 0.169) differed significantly with P=0.0058 (see Methods for testing interaction using GWAS summary statistics), suggesting PRS interacts with smoking status to increase lung adenocarcinoma risk with a stronger association in never-smokers.

Supplementary Table 1. The number of subjects stratified by smoking status and sex in East Asian populations.

	Discovery								Replication[a]	
	FLCCA (Female Lung Cancer Consortium in Asia)		NJLCS (Nanjing Lung Cancer Study, China)		NCC (National Cancer Center Research Institute, Japan)		ACC (Aichi Cancer Center, Japan)			
	Cases	Controls	Cases	Controls	Cases	Controls	Cases	Controls	Cases	Controls
Males			817	1537	1731	5995	721	694	4957	46494
Male never-smokers			316	890	327	2242	109	251	693	16102
Females with smoking history			66	33	305	1212	111	309	1151	11936
Female never-smokers	4438	4544	724	1084	1558	10461	530	1310	3104	45582
Total	4438	4544	1923	3544	3921	19910	1471	2564	9905	120114

[a]: The replication study in Japanese populations included cases from seven sources (BioBank Japan, NCC, Kanagawa Cancer Center, Akita University Hospital, Tokyo Medical and Dental University, Hospital and Gunma University Hospital, and Fukushima Medical University School of Medicine) and controls from BioBank Japan.

Supplementary Table 2. Meta-analysis in East Asian populations identified 14 SNPs with genome-wide significance, including two novel loci. All p-values are nominal and two-sided.

							All		Never-smokers		Smokers		Never-smoking females	
CHR	BP		Genes	SNP	Eff/Ref	EAF	OR	P	OR	P	OR	P	OR	P
5	1286516	5p15.33	*CMTPM1L,TERT*	rs2736100	A/C	0.39	0.75	7.9E-58	0.74	9.3E-44	0.77	1.1E-16	0.74	1.1E-36
10	114509390	10q25.2	*VTI1A*	rs11196089	C/T	0.30	1.26	6.7E-29	1.30	1.3E-25	1.18	4.2E-06	1.31	8.1E-24
3	189354377	3q28	*TP63*	rs55779747	C/A	0.47	0.82	8.5E-27	0.81	2.2E-20	0.84	4.7E-08	0.82	3.4E-17
6	117785558	6q22.1	*ROS1*, *DCBLD1*	rs6937083	T/A	0.48	0.85	1.9E-19	0.82	1.5E-18	0.91	0.00232	0.82	1.9E-16
6	41483640	6p21.1	*FOXP4*	rs9367106	C/G	0.34	1.20	1.1E-14	1.20	3.8E-10	1.21	5.8E-06	1.20	4.4E-09
6	32574608	6p21	*MHC*	rs2760995	A/G	0.13	1.19	1.1E-11	1.18	9.9E-08	1.20	2.9E-05	1.16	1.3E-05
6	30769565	6p21	*MHC*	rs9380190	C/T	0.30	0.87	1.4E-08	0.89	0.00031	0.85	6.7E-6	0.88	4.3E-04
17	65960754	17q24.2	*BPTF*	rs59956089	C/T	0.30	1.17	8.0E-11	1.18	3.6E-09	1.13	0.0045	1.18	1.5E-08
3	169514635	3q26.2	*LRRC34*	rs10936600	T/A	0.41	0.89	3.2E-10	0.88	2.4E-08	0.91	0.0026	0.88	5.3E-08
2	25757809	2p23.3	*DTNB*	rs682888[a]	C/T	0.47	0.89	4.9E-10	0.90	1.3E-06	0.88	8.9E-05	0.90	5.9E-06
7	124373484	7q31.33	*GPR37*	rs4268071[a]	T/G	0.04	1.39	7.3E-10	1.39	9.3E-06	1.40	1.9E-5	0.72	3.4E-05
12	52348509	12q13.13	*ACVR1B*	rs7962469	G/A	0.34	1.13	1.1E-09	1.16	3.2E-09	1.08	0.0347	1.17	2.4E-09
9	22160137	9p21.3	*CDKN2B*	rs72658409	T/C	0.09	0.79	1.8E-09	0.74	1.1E-09	0.89	0.095	0.75	3.3E-08
11	118104877	11q23.3	*AMICA1*	rs1573559[b]	A/C	0.42	0.90	4.3E-08	0.88	2.1E-07	0.93	0.039	0.89	2.9E-07

[a]: Novel SNPs.

[b]: rs1573559 was previously reported in EUR populations (McKay et al., Nature Genetics, 2017), but has never achieved genome-wide significance in EA.

Supplementary Table 3: Meta-analysis of three variants for lung adenocarcinoma combining the Chinese samples in Dai *et al.*[6] and our East Asian samples that are not overlapping with samples in Dai *et al.*[6]. These three variants were identified in a trans-ethnic analysis of Chinese samples and European samples in the ILCCO study. One variant, rs2293607, on chr3 achieved in genome-wide significance in the Chinese samples in Dai *et al.*[6]. The other two variants achieved genome-wide significance after combining the Chinese samples in Dai *et al.*[6] and our East Asian samples. All p-values are nominal and two-sided.

Chr	Base pair	SNP	Eff/ Ref	Trans-ethnic analysis in Dai *et al.*[6]		Chinese samples in Dai *et al.*[6] (6839 cases,9298 controls)		Meta-analysis of EA samples in our discovery samples, non-overlapping with Dai *et al.*[6] (10673 cases, 27241 controls)		Meta-analysis of East Asian samples: Chinese samples in Dai *et al.*[6] and our samples		
				OR (95% CI)	P	OR (95% CI)	P	OR (95% CI)	P	OR (95% CI)	P	P_{het}
2	65496058	rs17038564	G/A	1.15(1.20,1.21)	1.87E-08	1.15(1.09,1.21)	5.10E-07	1.09(1.04,1.15)	4.53E-04	1.12(1.08,1.16)	2.63E-09	0.15
3	169482335	rs2293607	C/T	0.88(0.85,0.91)	1.34E-15	0.86(0.83,0.90)	6.23E-12	0.90(0.86,0.94)	4.99E-07	0.88(0.85,0.91)	5.15E-17	0.13
14	35293185	rs1200399	T/C	0.89(0.86,0.92)	2.70E-10	0.89(0.85,0.93)	9.13E-08	0.91(0.87,0.95)	3.54E-06	0.90(0.87,0.93)	1.99E-12	0.45

Supplementary Table 4: Total of 28 susceptibility variants identified for LUAD in East Asian populations with genome-wide significance.

							All		Never-smokers		Smokers	
	CHR	BP	SNP	Genes	Eff/Ref	MAF	OR	P	OR	P	OR	P
Variants previously reported with genome-wide significance (GWS) in EA	5	1286516	rs2736100[7-13]	*CLPTM1L,TERT*	A/C	0.39	0.75(0.72,0.78)	7.92E-58	0.74(0.71,0.77)	9.33E-44	0.77(0.72,0.82)	1.12E-16
	10	114509290	rs11196089[7,10]	*VTI1A*	C/T	0.30	1.26(1.21,1.31)	6.72E-29	1.30(1.24,1.37)	1.27E-25	1.18(1.10,1.27)	4.19E-06
	3	189354127	rs55779747[7,8,10,11,12,13]	*TP63*	C/A	0.47	0.82(0.79,0.85)	8.47E-27	0.81(0.77,0.85)	2.15E-20	0.84(0.79,0.89)	4.67E-08
	6	117785308	rs6937083[7,10]	*ROS1/DCBLD1*	T/A	0.48	0.85(0.82,0.88)	1.95E-19	0.82(0.78,0.86)	1.46E-18	0.91(0.86,0.97)	0.00232
	3	169482335	rs2293607[6,a]	*LRRC34*	C/T	0.46	0.86(0.83,0.89)	2.89E-18				
	6	41483390	rs9367106[7,10]	*FOXP4*	C/G	0.34	1.20(1.15,1.26)	1.06E-14	1.20(1.13,1.27)	3.79E-10	1.21(1.11,1.31)	5.77E-06
	5	1280477	rs13167280[b]	*CLPTM1L-TERT*	A/G	0.22	1.29(1.20,1.38)	4.07E-13				
	6	32574358	rs2760995[7,10]	*MHC*	A/G	0.13	1.19(1.13,1.25)	1.08E-11	1.18(1.11,1.25)	9.87E-08	1.20(1.10,1.31)	2.87E-05
	17	65960854	rs59956089[10,12]	*BPTF*	C/T	0.30	1.17(1.12,1.23)	7.97E-11	1.18(1.12,1.25)	3.61E-09	1.13(1.04,1.23)	0.00449
	12	52348259	rs7962469[10,13]	*ACVR1B*	G/A	0.34	1.13(1.09,1.18)	1.13E-09	1.16(1.10,1.22)	3.23E-09	1.08(1.01,1.16)	0.03469
	9	22160087	rs72658409[10,13]	*CDKN2B-AS1*	T/C	0.09	0.79(0.73,0.85)	1.75E-09	0.74(0.67,0.82)	1.06E-09	0.89(0.78,1.02)	0.09553
	5	1290319	rs62332591[b]	*CLPTM1L-TERT*	G/T	0.48	0.87(0.83,0.91)	3.21E-08				
	6	30769565	rs9380190[7,10,12,13]	*MHC*	C/T	0.30	0.87(0.83,0.91)	1.40E-08	0.89(0.84,0.95)	3.10E-08	0.85(0.79,0.91)	6.70E-6
GWS in EUR	11	118108331	rs55768116[14]	*AMICA1*	A/C	0.42	0.90(0.87,0.93)	4.29E-08	0.88(0.84,0.92)	2.12E-07	0.93(0.87,1.00)	0.03929
GWS in multi-ancestry analysis but not in EA[c]	2	65496058	rs17038564[6]	*ACTR2*	G/A	0.20	1.12(1.08,1.16)	2.63E-09				
	14	35293185	rs1200399[6]	*BAZ1A*	T/C	0.50	0.90(0.87,0.93)	1.99E-12				
New variants achieving GWS in EA[e]	15	49757466	rs71467682[14,d]	*FGF7,SECISBP2L*	G/A	0.31	0.91(0.89,0.93)	2.81E-14	0.91(0.88,0.94)	4.17E-08	0.91(0.88,0.94)	9.71E-8
	3	138570011	rs137884934	*PIK3CB*	T/C	0.09	0.80(0.76,0.84)	6.21E-20	0.82(0.76,0.88)	2.97E-08	0.79(0.74,0.84)	2.21E-13
	2	25757709	rs682888	*DTNB*	C/T	0.47	0.90(0.88,0.92)	5.96E-19	0.90(0.87,0.93)	1.93E-11	0.91(0.88,0.94)	4.78E-09
	11	61581656	rs174559	*FADS1*	A/G	0.39	0.91(0.89,0.93)	1.93E-14	0.92(0.89,0.95)	2.87E-06	0.90(0.87,0.93)	6.01E-10
	10	126324209	rs10901793	*FAM53B,*	A/G	0.30	1.08(1.06,1.10)	3.04E-11	1.12(1.08,1.16)	9.21E-11	1.05(1.02,1.08)	2.90E-03
	6	41483960	rs12664490[b]	*FOXP4*	T/C	0.16	0.81(0.76,0.86)	1.21E-10				
	7	124373384	rs4268071	*GPR37*	T/G	0.04	1.39(1.25,1.54)	7.27E-10	1.38(1.19,1.60)	2.50E-05	1.32(1.15,1.51)	5.50E-05
	6	53389995	rs531557	*GCLC*	T/A	0.40	0.93(0.91,0.95)	9.25E-10	0.92(0.89,0.95)	1.07E-06	0.94(0.91,0.97)	1.52E-04
	19	725066	rs116863980	*PALM*	A/G	0.06	1.21(1.14,1.29)	2.63E-09	1.16(1.06,1.27)	1.00E-03	1.26(1.15,1.38)	2.97E-07
	15	56454223	rs764014	*RFX7*	G/A	0.47	0.94(0.91,0.96)	7.73E-09	0.91(0.88,0.94)	4.12E-08	0.96(0.93,0.99)	9.06E-03
	4	44174404	rs117715768	*KCTD8*	T/C	0.06	1.15(1.09,1.21)	2.45E-08	1.17(1.09,1.25)	8.72E-06	1.13(1.05,1.21)	5.42E-04
	4	157894892	rs1373058	*PDGFC*	A/T	0.43	1.07(1.05,1.10)	3.86E-08	1.08(1.04,1.12)	2.12E-05	1.07(1.03,1.11)	4.25E-04

[a]: The variant was first in reported in a multi-ancestry analysis of EA and EUR samples and has achieved GWS in Chinese samples in Dai *et al*.[6]. Reported OR and p-values are based on meta-analysis of Chinese samples in Dai *et al*.[6] and our EA samples.

[b]: Variants identified by conditional analysis.

[c]: The variants achieved GWS in a multi-ancestry meta-analysis of EA and EUR populations but did not achieve GWS in EA samples in Dai *et al*.[6]. The reported p-values in the table are based on meta-analysis of non-overlapping EA samples in Dai *et al*.[6] and our study. Details can be found in Supplementary Table 3.

[d]: Rs77468143 at this locus was previously reported only in EUR populations[14]. Rs71467682 reported in the table is in weak LD with rs77468143 (R2=0.27 in EA, R2=0 in EUR populations).

[e:] ORs and P-values are based on the combination of the discovery and replication data in our study except rs4268071 (replication not available).

All p-values are nominal and two-sided.

Supplementary Table 5. Comparing odds ratios (OR) of 28 risk variants of lung adenocarcinoma between Han Chinese and Japanese, between China mainland and others.

					Han Chinese vs. Japanese							China mainland vs. others						
					Han Chinese			Japanese			P-Het	China mainland			Out of China mainland			P-Het
CHR	BP	SNP	A1/A2	MAF	OR	SE	P	OR	SE	P		OR	SE	P	OR	SE	P	
2	25757709	rs682888	C/T	0.47	0.90	0.04	8.0E-05	0.91	0.02	8.0E-05	7.4E-01	0.87	0.05	1.6E-05	0.91	0.02	3.5E-06	4.1E-01
2	65496058	rs17038564	G/A	0.19	1.12	0.05	1.8E-03	1.08	0.03	8.3E-03	5.4E-01	1.11	0.06	1.6E-02	1.08	0.03	3.1E-03	7.0E-01
3	138570011	rs137884934	T/C	0.10	0.85	0.08	5.0E-02	0.81	0.06	2.5E-04	6.1E-01	0.84	0.11	1.1E-01	0.82	0.05	5.8E-05	8.1E-01
3	169482335	rs2293607	C/T	0.47	0.92	0.04	2.8E-02	0.90	0.02	2.2E-05	5.7E-01	0.93	0.05	1.6E-01	0.90	0.02	1.9E-06	5.2E-01
3	189354127	rs55779747	C/A	0.48	0.82	0.04	8.6E-13	0.82	0.02	2.5E-16	9.3E-01	0.83	0.05	1.0E-08	0.82	0.02	1.1E-21	8.2E-01
4	44174404	rs117715768	T/C	0.08	1.20	0.07	1.2E-02	1.23	0.05	6.1E-05	7.7E-01	1.19	0.10	7.1E-02	1.24	0.05	3.4E-06	7.5E-01
4	157894892	rs1373058	A/T	0.40	1.07	0.04	7.6E-02	1.11	0.03	9.5E-05	4.1E-01	1.15	0.05	6.2E-03	1.09	0.02	1.5E-04	3.7E-01
5	1280477	rs13167280	A/G	0.23	1.57	0.05	9.4E-17	1.52	0.04	1.2E-21	6.4E-01	1.48	0.07	5.7E-08	1.47	0.04	1.7E-25	9.5E-01
5	1286516	rs2736100	A/C	0.45	0.72	0.04	7.0E-32	0.75	0.02	4.3E-33	3.5E-01	0.75	0.05	4.2E-18	0.73	0.02	4.2E-48	6.3E-01
5	1290319	rs62332591	G/T	0.47	0.78	0.05	1.3E-07	0.78	0.03	2.2E-19	9.9E-01	0.83	0.06	3.5E-03	0.77	0.03	2.7E-24	3.1E-01
6	30769565	rs9380190	C/T	0.42	0.90	0.05	2.3E-03	0.89	0.03	1.1E-05	7.6E-01	0.88	0.07	4.6E-04	0.90	0.03	4.0E-05	7.0E-01
6	32574358	rs2760995	A/G	0.14	1.15	0.05	3.8E-04	1.20	0.03	1.1E-07	5.9E-01	1.17	0.07	5.5E-04	1.18	0.03	2.1E-08	8.6E-01
6	41483390	rs9367106	C/G	0.32	1.16	0.05	2.6E-03	1.22	0.03	1.2E-12	3.4E-01	1.19	0.07	9.0E-03	1.2	0.03	4.4E-13	8.8E-01
6	41483960	rs12664490	T/C	0.16	0.78	0.06	4.4E-05	0.81	0.04	9.5E-08	5.8E-01	0.73	0.08	1.4E-04	0.81	0.03	3.7E-09	2.4E-01
6	53389995	rs531557	T/A	0.42	0.87	0.04	1.8E-04	0.91	0.03	1.7E-04	3.5E-01	0.90	0.05	2.6E-02	0.9	0.02	1.9E-06	9.4E-01
6	117785308	rs6937083	T/A	0.48	0.85	0.04	1.4E-09	0.85	0.02	3.6E-11	8.4E-01	0.86	0.05	2.1E-06	0.85	0.02	4.0E-15	8.3E-01
7	124373384	rs4268071	G/T		0.66	0.23	7.0E-02	0.74	0.05	3.9E-09	6.6E-01	0.60	0.28	6.8E-02	0.74	0.05	6.2E-09	4.6E-01
9	22160087	rs72658409	T/C	0.07	0.77	0.07	3.2E-06	0.83	0.06	1.3E-03	4.4E-01	0.79	0.09	3.6E-04	0.79	0.05	1.2E-06	9.5E-01
10	114509290	rs11196089	C/T	0.31	1.24	0.04	2.5E-12	1.25	0.03	2.7E-15	8.8E-01	1.21	0.05	1.7E-07	1.27	0.02	4.9E-23	3.9E-01
10	126324209	rs10901793	A/G	0.31	1.13	0.04	6.8E-05	1.09	0.02	2.7E-04	5.2E-01	1.11	0.05	2.3E-03	1.1	0.02	5.9E-06	8.8E-01
11	61581656	rs174559	A/G	0.47	0.88	0.04	1.7E-05	0.92	0.03	6.5E-04	3.9E-01	0.89	0.05	5.8E-04	0.92	0.02	1.0E-04	5.6E-01
11	118108331	rs55768116	A/C	0.47	0.88	0.04	4.2E-04	0.90	0.02	2.1E-05	6.6E-01	0.88	0.05	7.7E-03	0.9	0.02	4.6E-07	7.2E-01

12	52348259	rs7962469	G/A	0.33	1.17	0.04	3.4E-05	1.13	0.02	2.1E-06	3.9E-01	1.23	0.05	3.2E-05	1.13	0.02	2.4E-08	1.1E-01
14	35293185	rs1200399	T/C	0.49	0.94	0.04	1.6E-02	0.88	0.02	4.0E-07	1.9E-01	0.90	0.05	2.0E-03	0.91	0.02	9.4E-06	9.1E-01
15	49757466	rs71467682	G/A	0.31	0.91	0.04	1.4E-03	0.91	0.03	3.5E-04	9.6E-01	0.90	0.05	2.9E-03	0.91	0.02	2.9E-05	9.2E-01
15	56454223	rs764014	G/A	0.41	0.88	0.04	1.1E-05	0.92	0.02	2.7E-04	4.0E-01	0.89	0.05	3.8E-04	0.92	0.02	9.6E-05	4.9E-01
17	65960854	rs59956089	C/T	0.31	1.14	0.04	1.6E-03	1.19	0.03	7.4E-09	3.7E-01	1.10	0.05	6.7E-02	1.18	0.03	8.5E-11	2.4E-01
19	725066	rs116863980	A/G	0.06	1.26	0.10	2.1E-02	1.31	0.08	3.0E-04	7.3E-01	1.17	0.14	2.6E-01	1.33	0.06	1.2E-05	3.9E-01

Supplementary Table 6: Association between new SNPs and smoking behaviors. Data were extracted from the BioBank Japan (http://jenger.riken.jp/en/). All p-values are nominal and two-sided.

					Age of initiation (N=30418)		Cigarettes per day (N=72655)		Ever smoked (N=83830 ever, N=81626 never)		Quit (N=32884 current; N=43163 former)		Chronic obstructive pulmonary disease (N=3315 cases, N=201592 controls)	
Chr	BP	SNP	Genes	Eff/Ref	Beta	P	Beta	P	Beta	P	Beta	P	Beta	P
2	25757709	rs682888	*DTNB*	C/T	0.001	0.95	0.009	0.09	0.001	0.62	0.001	0.76	0.028	0.276
3	138570011	rs137884934	*PIK3CB*	T/C										
4	44174404	rs117715768	*KCTD8*	T/C									0.150	0.013
4	157894892	rs1373058	*PDGFC*	A/T	0.018	0.05	0.002	0.77	-0.002	0.20	-0.001	0.66	0.012	0.662
6	53389995	rs531557	*GCLC*	T/A	0.001	0.94	0.002	0.69	-0.001	0.74	0.001	0.69	-0.019	0.489
7	124373384	rs4268071	*GPR37*	G/T	-0.030	0.09	-0.027	0.02	0.006	0.06	-0.003	0.61	-0.02915	0.61
10	126324209	rs10901793	*FAM53B, METTL10*	A/G	0.017	0.05	-0.001	0.81	0.001	0.36	-0.002	0.51	-0.016	0.537
11	61581656	rs174559	*FADS1*	A/G	0.003	0.72	-0.008	0.14	0.000	0.93	0.001	0.67	0.027	0.304
15	49757466	rs71467682	*SECISBP2L*	G/A	0.005	0.61	-0.002	0.71	-0.001	0.67	-0.005	0.05	-0.019	0.505
15	56454223	rs764014	*RFX7*	G/A	0.011	0.17	-0.004	0.49	0.000	0.97	0.002	0.39	0.007	0.793
19	725066	rs116863980	*PALM*	A/G										

Supplementary Table 7. Multi-ancestry meta-analysis of East Asian (EA) and European (EUR) GWAS.

					Multi-ancestry meta-analysis		EA			EUR		
	Chr	BP	SNP	Eff/Ref	OR	P	EAF	OR	P	EAF	OR	P
Novel loci	2	85893741	rs1130866[a]	A/G	1.08	1.56E-08	0.28	1.08	8.10E-05	0.52	1.07	1.35E-05
	4	164070122	rs2320614	C/T	1.08	6.51E-09	0.73	1.06	5.58E-03	0.40	1.09	2.48E-07
	16	82153538	rs34638657	C/A	1.09	2.19E-09	0.14	1.09	8.52E-04	0.23	1.09	6.39E-06
	18	29922921	rs638868	C/A	1.08	3.60E-08	0.21	1.09	2.91E-05	0.21	1.07	2.43E-04
New SNPs at existing loci	6	53383788	rs61583240[b]	T/C	1.12	3.66E-08	0.08	1.16	7.90E-06	0.12	1.10	5.25E-04
	20	62314054	rs75031349[c]	G/A	0.86	6.38E-09	0.04	0.81	2.00E-04	0.07	0.87	7.68E-06

[a]: rs1130866 is an isolated SNP with no SNPs in LD. This SNP was genotyped for all studies in EA GWAS and in the ILCCO GWAS[14].

[b]: Our East Asian analysis identified rs531557. SNP rs61583240 identified by trans-ethnic analysis is in weak LD with rs531557 ($R2=0.15$ in EA and $R2=0.08$ in EUR). However, OR=1.09 and P=8.85E-5 after conditioning on rs531557. Thus, this SNP was not considered as novel variant genome-wide significance.

[c]: McKay *et al.*[14] reported rs41309931 for LUAD. SNP rs75031349 identified here is in very weak LD with rs41309931 ($R2=0$ in EA and $R2=0.006$ in EUR). Conditional analysis (on rs41309931) led to an attenuated association with OR=0.88 and P=4.0E-7. This SNP was not considered as novel variant genome-wide significance.

All p-values are nominal and two-sided.

Supplementary Table 8: Sample sizes of GWAS in East Asian and European populations

	East Asian		European	
	Cases	Controls	Cases	Controls
Smokers	3,751	9,780	6137[a]	9084[a]
Nonsmokers	8,002	20,782	2509[b]	6296[b]
Total	11,753	30,562	11273[c]	55483[c]

[a]: We only used subjects genotyped on the OncoArray platform from the ILCCO study[14] for analyses restricted to smokers. Data were not available for LUAD risk stratified by smoking status for studies that had not been genotyped on this platform.

[b]: Subjects were from the Hung *et al.*[15] GWAS of LUAD among never-smokers.

[c]: All LUAD cases and controls in McKay *et al.*[14] were included for analyses that combined smokers and nonsmokers. Note that, "c" has more smokers than "a" but fewer never-smokers than "b". For any comparison using smokers + nonsmokers, we used "c" to maximize the power.

Supplementary Table 9. Mendelian randomization (MR) analysis using MR PRESSO[16] were based on 46 variants that achieved genome-wide significance in the telomere GWAS in the TOPMed[17] study combining samples of all ethnic populations. The original paper identified 48 variants associated with telomere length that collectively explained 4.35% of telomere length variance; two of them at the *TERT* locus were excluded using the LD filter $R^2<0.05$ that together explained 0.61% of the telomere length variance; the remaining 46 variants included in MR analysis explained 3.74% of telomere length variance. All p-values are two-sided and nominal.

MR Analysis in EA populations				MR Analysis in EUR populations			
Genetic effects of instruments based on:	LUAD GWAS	OR (95% CI)	p	Genetic effects of instruments based on:	LUAD GWAS	OR (95% CI)	p
All TOPMed samples	All	2.61 (2.08, 3.28)	8.14E-10	All TOPMed samples	All	2.67 (2.07, 3.43)	7.14E-09
	Smokers	1.83 (1.32, 2.52)	7.44E-04		Smokers	2.92 (2.16, 3.94)	2.88E-08
	Nonsmokers	2.89 (2.20, 3.81)	5.75E-09		Nonsmokers	2.36 (1.56, 3.56)	2.17E-04
Asian samples in TOPMed	All	2.17 (1.72, 2.74)	1.59E-07	EUR samples in TOPMed	All	3.79 (2.70, 5.33)	3.05E-09
	Smokers	1.62 (1.15, 2.27)	8.98E-03		Smokers	3.97 (2.87, 5.51)	3.93E-10
	Nonsmokers	2.40 (1.81, 3.19)	5.50E-07		Nonsmokers	3.87 (2.40, 6.26)	1.80E-06

Supplementary References

1. Bulik-Sullivan B, *et al.* An atlas of genetic correlations across human diseases and traits. *Nature Genetics* **47**, 1236-1241 (2015).
2. Moyerbrailean GA, *et al.* High-throughput allele-specific expression across 250 environmental conditions. *Genome Res* **26**, 1627-1638 (2016).
3. Bosse Y, *et al.* Molecular signature of smoking in human lung tissues. *Cancer Res* **72**, 3753-3763 (2012).
4. Beane J, *et al.* Characterizing the impact of smoking and lung cancer on the airway transcriptome using RNA-Seq. *Cancer Prev Res (Phila)* **4**, 803-817 (2011).
5. Brown BC, Asian Genetic Epidemiology Network Type 2 Diabetes C, Ye CJ, Price AL, Zaitlen N. Transethnic Genetic-Correlation Estimates from Summary Statistics. *Am J Hum Genet* **99**, 76-88 (2016).
6. Dai J, *et al.* Identification of risk loci and a polygenic risk score for lung cancer: a large-scale prospective cohort study in Chinese populations. *Lancet Respir Med* **7**, 881-891 (2019).
7. Lan Q, *et al.* Genome-wide association analysis identifies new lung cancer susceptibility loci in never-smoking women in Asia. *Nat Genet* **44**, 1330-1335 (2012).
8. Hu Z, *et al.* A genome-wide association study identifies two new lung cancer susceptibility loci at 13q12.12 and 22q12.2 in Han Chinese. *Nat Genet* **43**, 792-796 (2011).
9. Hsiung CA, *et al.* The 5p15.33 locus is associated with risk of lung adenocarcinoma in never-smoking females in Asia. *PLoS Genet* **6**, (2010).
10. Seow WJ, *et al.* Association between GWAS-identified lung adenocarcinoma susceptibility loci and EGFR mutations in never-smoking Asian women, and comparison with findings from Western populations. *Hum Mol Genet* **26**, 454-465 (2017).
11. Miki D, *et al.* Variation in TP63 is associated with lung adenocarcinoma susceptibility in Japanese and Korean populations. *Nat Genet* **42**, 893-896 (2010).
12. Shiraishi K, *et al.* A genome-wide association study identifies two new susceptibility loci for lung adenocarcinoma in the Japanese population. *Nat Genet* **44**, 900-903 (2012).
13. Wang Z, *et al.* Meta-analysis of genome-wide association studies identifies multiple lung cancer susceptibility loci in never-smoking Asian women. *Hum Mol Genet* **25**, 620-629 (2016).
14. McKay JD, *et al.* Large-scale association analysis identifies new lung cancer susceptibility loci and heterogeneity in genetic susceptibility across histological subtypes. *Nat Genet* **49**, 1126-1132 (2017).
15. Hung RJ, *et al.* Lung Cancer Risk in Never-Smokers of European Descent is Associated With Genetic Variation in the 5p15.33 TERT-CLPTM1Ll Region. *J Thorac Oncol* **14**, 1360-1369 (2019).
16. Verbanck M, Chen CY, Neale B, Do R. Publisher Correction: Detection of widespread horizontal pleiotropy in causal relationships inferred from Mendelian randomization between complex traits and diseases. *Nat Genet* **50**, 1196 (2018).
17. Margaret A. Taub MPC, Rebecca Keener, Kruthika R. Iyer, Joshua S. Weinstock, Lisa R. Yanek, John Lane, Tyne W. Miller-Fleming, Jennifer A. Brody, Laura M. Raffield,

Caitlin P. McHugh, Deepti Jain, Stephanie M. Gogarten, Cecelia A. Laurie, et al. Novel genetic determinants of telomere length from a trans-ethnic analysis of 109,122 whole genome sequences in TOPMed. *Cell Genomics* **2**, 100084 (2022).
