# Supplementary material for: Genome-wide association study of lung adenocarcinoma in East Asia and comparison with a European population
Source: Nat Commun. 2023 May 26;14:3043. doi: 10.1038/s41467-023-38196-z (PMC10220065; doi:10.1038/s41467-023-38196-z)
Supplement: Supplementary file 3 — Description of Additional Supplementary Files [file 41467_2023_38196_MOESM3_ESM.pdf]

Description of Additional Supplementary Files for "Genome-wide association study of lung adenocarcinoma in East Asia and comparison with a European population".

File name: Supplementary Data 1
Description: Fine-mapping results of significant risk loci from the East Asian discovery set using FINEMAP.

File name: Supplementary Data 2
Description: Functional annotation of proxy variants R2 > 0.8 with the lead SNP for 12 novel signals from the GWAS of East Asian populations.

File name: Supplementary Data 3
Description: Stratified LD score regression analysis investigating functional enrichment and relevant tissues for LUAD heritability.

File name: Supplementary Data 4
Description: cis-eQTL of GWAS variants in Taiwanese lung and GTEx v8 lung datasets

File name: Supplementary Data 5
Description: Taiwanese lung eQTL genes colocalizing with LUAD GWAS

File name: Supplementary Data 6
Description: TWAS genes using Taiwanese eQTL dataset (LCTCNS) or GTEx v8 lung eQTL dataset with the East Asian discovery GWAS summary stats

File name: Supplementary Data 7
Description: Pathways enriched in lung cancer GWAS genes by IPA

File name: Supplementary Data 8
Description: Comparison of risk SNPs between East Asian and European populations

File name: Supplementary Data 9
Description: Power calculation for SNPs that show significant heterogeneity

File name: Supplementary Data 10
Description: Meta-analysis results in East Asian populations for SNPs with p-value ≤0.01

File name: Supplementary Data 11
Description: The results of the replication study for the 38 SNPs in East Asian populations.

File name: Supplementary Data 12
Description: The GWAS summary data for SNPs with p<0.01 in the FLCCA study and SNPs with genome-wide significance in the meta-analysis of East Asian samples.

File name: Supplementary Data 13
Description: The GWAS summary data for SNPs with $p<0.01$ in the NJLCS study and SNPs with genome-wide significance in the meta-analysis of East Asian samples.
