# Supplementary material for: Genome-wide association study of lung adenocarcinoma in East Asia and comparison with a European population
Source: Nat Commun. 2023 May 26;14:3043. doi: 10.1038/s41467-023-38196-z (PMC10220065; doi:10.1038/s41467-023-38196-z)
Supplement: Supplementary file 17 — Reporting Summary [file 41467_2023_38196_MOESM17_ESM.pdf]

nature portfolio

Corresponding author(s): Qing Lan

Last updated by author(s): Mar 18, 2022

Reporting Summary

Nature Portfolio wishes to improve the reproducibility of the work that we publish. This form provides structure for consistency and transparency in reporting. For further information on Nature Portfolio policies, see our Editorial Policies and the Editorial Policy Checklist.

Statistics

For all statistical analyses, confirm that the following items are present in the figure legend, table legend, main text, or Methods section.

n/a	Confirmed	
☐	☒	The exact sample size (*n*) for each experimental group/condition, given as a discrete number and unit of measurement
☒	☐	A statement on whether measurements were taken from distinct samples or whether the same sample was measured repeatedly
☐	☒	The statistical test(s) used AND whether they are one- or two-sided<br>*Only common tests should be described solely by name; describe more complex techniques in the Methods section.*
☐	☒	A description of all covariates tested
☐	☒	A description of any assumptions or corrections, such as tests of normality and adjustment for multiple comparisons
☐	☒	A full description of the statistical parameters including central tendency (e.g. means) or other basic estimates (e.g. regression coefficient) AND variation (e.g. standard deviation) or associated estimates of uncertainty (e.g. confidence intervals)
☐	☒	For null hypothesis testing, the test statistic (e.g. *F*, *t*, *r*) with confidence intervals, effect sizes, degrees of freedom and *P* value noted<br>*Give P values as exact values whenever suitable.*
☒	☐	For Bayesian analysis, information on the choice of priors and Markov chain Monte Carlo settings
☒	☐	For hierarchical and complex designs, identification of the appropriate level for tests and full reporting of outcomes
☐	☒	Estimates of effect sizes (e.g. Cohen's *d*, Pearson's *r*), indicating how they were calculated

Our web collection on statistics for biologists contains articles on many of the points above.

Software and code

Policy information about availability of computer code

Data collection	No software was used for data collection.
Data analysis	HyPrColoc (https://github.com/jrs95/hyprcoloc); eCAVIAR v2.0.0 (http://genetics.cs.ucla.edu/caviar/); ezQTL (https://analysistools.cancer.gov/ezqtl/#/home); FORGE2 (https://forge2.altiusinstitute.org); FUSION (http://gusevlab.org/projects/fusion/); FINEMAP V1.4.1 (http://www.christianbenner.com/); minimac V4.0.3 (https://github.com/statgen/Minimac4); GENESIS (https://github.com/yandorazhang/GENESIS); HaploReg v4.1 (https://pubs.broadinstitute.org/mammals/haploreg/haploreg.php); Haploreg(https://pubs.broadinstitute.org/mammals/haploreg/haploreg_v4.php); LDSC V1.0.1 (https://github.com/bulik/ldsc); minimac4 (https://github.com/statgen/Minimac4); MR-PRESSO (https://github.com/rondolab/MR-PRESSO); GCTA https://cnsgenomics.com/software/gcta/#Overview); PLINK V2.0 (https://www.cog-genomics.org/plink/2.0/); POPCORN (https://github.com/brielin/Popcorn); IMPUTE2 V2 (https://mathgen.stats.ox.ac.uk/impute/impute_v2.html); R x64 4.1.0 (https://www.r-project.org/); RegulomeDB v2.1 (https://regulomedb.org/regulome-search/); SNPTEST V2 (https://mathgen.stats.ox.ac.uk/genetics_software/snptest/old/snptest.html).

For manuscripts utilizing custom algorithms or software that are central to the research but not yet described in published literature, software must be made available to editors and reviewers. We strongly encourage code deposition in a community repository (e.g. GitHub). See the Nature Portfolio guidelines for submitting code & software for further information.

Data

Policy information about availability of data

All manuscripts must include a data availability statement. This statement should provide the following information, where applicable:

- Accession codes, unique identifiers, or web links for publicly available datasets
- A description of any restrictions on data availability
- For clinical datasets or third party data, please ensure that the statement adheres to our policy

All data supporting the findings described in this manuscript are available in the article and in the Supplementary Information and from the corresponding author or as otherwise indicated upon request. Full TWAS results are included in Supplementary Data 6. The summary statistics for the meta-analysis of the 4 GWAS datasets in East Asian populations for SNPs with p≤0.01 are in Supplementary Data 10. The results of the replication study for the 38 SNPs tested and the meta-analysis with the GWAS data are in Supplementary Data 11. For the FLCCA study, the GWAS summary data for SNPs with p<0.01 in the study and all SNPs with genome-wide significance in the meta-analysis of East Asian samples are in Supplementary Data 12. The individual genotype data for the FLCCA data are in dbGaP phs000716.v1.p1 (Genome-Wide Association Study of Lung Cancer Susceptibility in Never-Smoking Women in Asia). For the NJLCS study, the GWAS summary data for SNPs with p<0.01 in the study and all SNPs with genome-wide significance in the meta-analysis of East Asian samples are in Supplementary Data 13. For the NCC and ACC studies, please contact Kouya Shiraishi at kshirais@ncc.go.jp or Takashi Kohno at tkkohno@ncc.go.jp for summary statistics. The GWAS data for the European populations contributing to this study are available at dbGap under accession phs000877.v1.p1 (Transdisciplinary Research Into Cancer of the Lung (TRICL), https://www.ncbi.nlm.nih.gov/projects/gap/cgi-bin/study.cgi?study_id=phs000876.v2.p1), phs001273.v3.p2 (Oncoarray Consortium, https://www.ncbi.nlm.nih.gov/projects/gap/cgi-bin/study.cgi?study_id=phs001273.v3.p2). To gain access to all data in dbGaP cited in this paper, please apply for dbGaP Authorized Access. The expression data of the lung cancer tissue cohort of never-smokers in Taiwan are publicly available at Gene Expression Omnibus under accession number GSE46539 https://www.ncbi.nlm.nih.gov/geo/query/acc.cgi?acc=GSE46539. The expression and eQTL data from GTEx (v6 and v8) are available from https://gtexportal.org/home/datasets.

Field-specific reporting

Please select the one below that is the best fit for your research. If you are not sure, read the appropriate sections before making your selection.

☒ Life sciences ☐ Behavioural & social sciences ☐ Ecological, evolutionary & environmental sciences

For a reference copy of the document with all sections, see nature.com/documents/nr-reporting-summary-flat.pdf

Life sciences study design

All studies must disclose on these points even when the disclosure is negative.

Sample size	For East Asian populations, the discovery data included 11,753 cases and 30,562 controls; the replication data included 9,905 cases and 120,114. For the European populations, the GWAS data included 11,273 cases and 55,483 controls. Sample sizes were determined using all GWAS data that are available to the project.
Data exclusions	The quality control filters for samples and genetic variants are described in Methods. Genetic variants were excluded based on genotyping call rate, minor allele frequency, Hardy-Weinberg equilibrium, and imputation quality score. Samples were excluded based on sample call rate, sex discrepancy, and relatedness.
Replication	Novel variants identified in the East Asian GWAS meta-analysis were replicated in an independent Japanese population with 9905 cases and 120,114 controls.
Randomization	This is population-based case-control study and randomization is relevant to this study.
Blinding	This is a population-base case-control study and blinding is relevant to the study.

Reporting for specific materials, systems and methods

We require information from authors about some types of materials, experimental systems and methods used in many studies. Here, indicate whether each material, system or method listed is relevant to your study. If you are not sure if a list item applies to your research, read the appropriate section before selecting a response.

Materials & experimental systems

n/a	Involved in the study
☒	☐ Antibodies
☒	☐ Eukaryotic cell lines
☒	☐ Palaeontology and archaeology
☒	☐ Animals and other organisms
☐	☒ Human research participants
☒	☐ Clinical data
☒	☐ Dual use research of concern

Methods

n/a	Involved in the study
☒	☐ ChIP-seq
☒	☐ Flow cytometry
☒	☐ MRI-based neuroimaging

Human research participants

Policy information about studies involving human research participants

Population characteristics

In the discovery data for the East Asians, the case group included 66% females and 34% females, 32% smokers and 68% never smokers; the control group included 38% males and 62% females, 32% smokers and 68% never smokers. In the replication data for East Asians, the case group included 57% females and 43% females, 62% smokers and 38% never smokers; the control group included 52% males and 48% females, 49% smokers and 51% never smokers.

Recruitment

Subjects were recruited through case-control and cohort studies, hospitals and clinics. The discovery data of East Asians and the data of Europeans were from the previously published GWAS of lung adenocarcinoma. The replication study included cases from multiple sources (BBJ, NCC, Kanagawa Cancer Center, Akita University Hospital, Tokyo Medical and Dental University, Hospital and Gunma University Hospital, and Fukushima Medical University School of Medicine) and non-cancer controls from BioBank Japan.

Ethics oversight

All participants provided informed consent according to protocols that were evaluated and approved by the internal review boards of the contributing centers. Protocols used to generate new, unpublished data presented in this paper were approved by the National Cancer Center Institutional Review Board, Japan and the Aichi Cancer Center Ethics Committee, Japan.

Note that full information on the approval of the study protocol must also be provided in the manuscript.
